# Combining triglyceride glucose-body mass index and high-sensitivity C-reactive protein to predict cardiovascular disease: results from a national cohort and a clinical verified cohort

**DOI:** 10.3389/fcvm.2025.1726615

**Published:** 2026-01-07

**Authors:** Wenheng Liu, Shu Zhang

**Affiliations:** Department of Cardiology, Fuwai Hospital, National Center for Cardiovascular Diseases, Chinese Academy of Medical Sciences and Peking Union Medical College, Beijing, China

**Keywords:** cardiovascular disease, China health and retirement longitudinal study, high-sensitivity C-reactive protein, inflammation, insulin resistance, triglyceride glucose-body mass index

## Abstract

**Background:**

Accumulating evidence highlights a strong association between insulin resistance (IR) and inflammation in the context of cardiovascular disease (CVD). However, the combined impact of both factors on the risk of developing CVD remains underexplored. This study aims to investigate how inflammation and insulin resistance interact to influence the risk of incident CVD.

**Methods:**

A total of 6,534 adults from the CHARLS were included. The primary endpoint was the occurrence of cardiovascular disease (CVD), defined as a composite of stroke and cardiac events. Participants were categorized according to the median values of two key markers: the triglyceride glucose-body mass index (TyG-BMI index) and high-sensitivity C-reactive protein (hsCRP). Cox proportional hazard models assessed the associations between TyG-BMI index, hsCRP, and the endpoints. To validate the reliability of the results, we clinically enrolled 805 patients who had undergone percutaneous coronary intervention (PCI).

**Results:**

In CHARLS, Multivariate Cox regression analysis revealed a significant association between elevated TyG-BMI index and hsCRP levels and an increased risk of CVD. Participants in the high TyG-BMI and high hsCRP group demonstrated a substantially higher risk of CVD (HR: 1.38, 95% CI: 1.18–1.62, *P* < 0.001) compared to those with low TyG-BMI and low hsCRP. Restricted cubic spline (RCS) analysis indicated a non-linear, positive relationship between both the TyG-BMI index and hsCRP levels with the risk of developing CVD. In the validation cohort, we also observed that the high TyG-BMI group had higher risks of MACCE, stroke, and cardiac events (all *P* < 0.05), and that the predictive performance of the model was significantly improved after adding TyG-BMI to the baseline model.

**Conclusion:**

These results emphasize the importance of simultaneously considering both TyG-BMI index and hsCRP levels in risk stratification for CVD, highlighting their combined potential to enhance early identification of high-risk individuals. Importantly, TyG-BMI also demonstrated significant predictive value for adverse outcomes in patients after PCI.

## Introduction

Despite significant progress in preventing, diagnosing, and treating cardiovascular disease (CVD), it still stands as the leading cause of premature death globally, accounting for approximately 19 million deaths in 2020, posing an increasingly burden of public health and societal productivity (1, 2). Numerous well-established risk factors for CVD include age, male sex, hypertension, diabetes mellitus (DM), abdominal obesity, and dyslipidemia. However, individuals developing CVD without these risk factors are not uncommon in clinical practice (3, 4), suggesting the existence of residual cardiovascular risk, such as inflammation, lipoprotein(a), remnant cholesterol, and insulin resistance (IR). Hence, early identification of individuals with residual cardiovascular risk holds significant clinical importance for optimizing risk stratification and primary prevention of CVD (5).

Insulin resistance, defined as the failure of insulin-dependent cells responding normally for insulin (6), plays a crucial role in the initiation and progression of DM and atherosclerosis (7–9). Growing evidence demonstrates a positive association between IR and CVD in individuals, whether with or without DM (10). Furthermore, a recent Mendelian randomization analysis has confirmed the causal relationship between IR and CVD (11). While the hyperinsulinemic-euglycemic clamp is considered the gold-standard method to identify (12), promoting it in clinical practice poses considerable difficulties due to its high cost, invasive nature, and complex procedure. Hence, the triglyceride-glucose (TyG) index has been proposed as a reliable surrogate indicator of IR, given its strong correlation with the gold standard and its convenience for clinical practice and large epidemiological studies (13, 14). Therefore, the TyG index is considered a reliable and effective predictor for the occurrence or outcome of CVD (5). Obesity, quantified by body mass index (BMI), is recognized as a major factor in IR (15). Recent studies have suggested that the TyG-BMI index, combining the TyG index with BMI, is superior to other parameters, such as the TyG index, for assessing IR in both the Korean and Chinese populations (16, 17).

Atherosclerosis is considered a chronic inflammatory disease, and accumulating evidence suggests that low-grade chronic inflammation could promote the progression of CVD (18, 19). Previous studies have shown that hsCRP > 2.0 mg/L is significantly associated with a higher risk of CVD and all-cause death, even in individuals with an ideal level of low-density lipoprotein (LDL) (20, 21). Similarly, a reduced systemic inflammation burden, as reflected by the level of hsCRP, is associated with better outcomes in patients undergoing lipid-lowering therapy (22–24).

Interestingly, several recent studies have reported a positive association between IR and systemic inflammation, particularly the level of hsCRP. Elevated systemic inflammation may contribute to IR (25, 26). However, to date, few studies have investigated the combined effects of IR and systemic inflammation on the risk of CVD. Therefore, we aimed to evaluate the individual and combined value of the TyG-BMI index and hsCRP in estimating the risk of CVD using data from CHARLS in the present study.

## Methods

### Study design and population

We conducted the study by extracting data from CHARLS (available at http://charls.pku.edu.cn/en), a prospective nationwide cohort study involving residents in rural and urban areas of China who are aged ≥45 years (27) (detailed in the Supplementary Methods).

**Figure 1:**
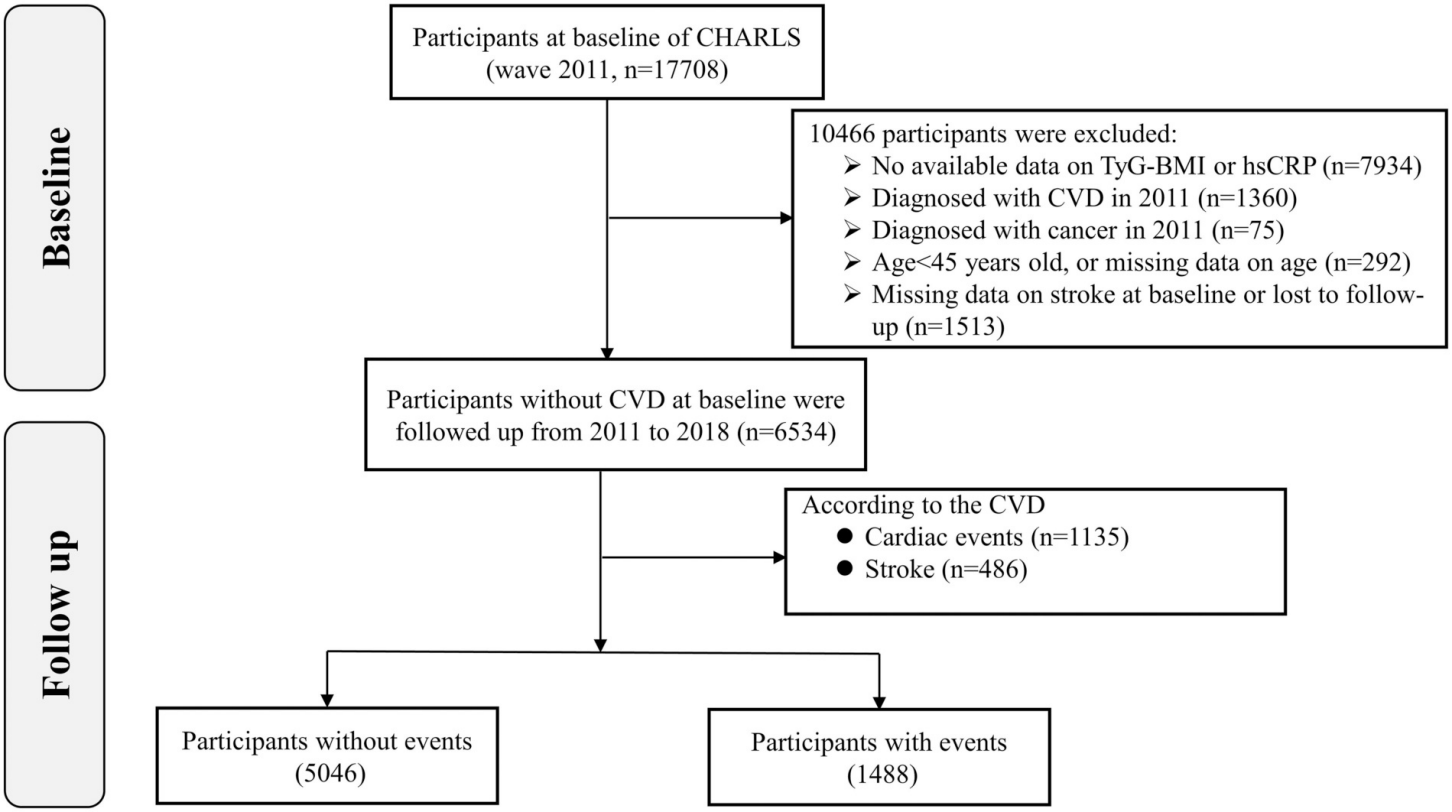
The flowchart of study participants.

Of the 17,708 participants in 2011, 6,534 individuals were ultimately included in this study based on selective criteria (Figure 1): (1) having available information on triglyceride (TG), fasting blood glucose (FBG), and BMI to calculate the TyG-BMI; (2) having available data on hsCRP; (3) aged ≥ 45 years; (4) without self-reported stroke or cardiac events in 2011; (5) without a history of cancer at baseline; (6) without missing data/unknown status regarding stroke or cardiac events, or loss or death in follow-up. The enrolled participants underwent follow-up every 1–2 years until 2018. The study population was classified into low TyG-BMI and high TyG-BMI, or low hsCRP and high hsCRP, based on the median of the TyG-BMI index or the levels of hsCRP (<2 mg/L or ≥2 mg/L) (28), and further divided into four groups according to the discordant/concordant TyG-BMI and hsCRP: low TyG-BMI and low hsCRP (Group 1); high TyG-BMI and low hsCRP (Group 2); low TyG-BMI and high hsCRP (Group 3); high TyG-BMI and high hsCRP (Group 4).

The data used in the study were obtained from a public dataset (https://doi.org/10.5061/dryad.13d31) uploaded by Yao HM et al. (29). This study with a waiver of informed consent has obtained the approval from the ethics committee of the First Affiliated Hospital of Zhengzhou University. Considering the nature of public dataset, no further research ethic was needed in the present study. The detailed study design has been described by Yao HM et al. (29). In our study, we selected participants aged 60 years or older with coronary artery disease who had undergone PCI as the validation cohort.

### Ascertainment of exposure

BMI (kg/m^2^) was computed according to the following formula: BMI = body mass/height^2^. Subsequently, the TyG-BMI index was calculated as: TyG-BMI = ln [TG (mg/dL) × FBG (mg/dL)] × BMI (kg/m^2^). The level of hsCRP were determined by Immunoturbidimetric assay.

### Outcome assessment

In CHARLS, the primary outcome was CVD, including stroke or cardiac events, and the secondary outcomes were stroke and cardiac events, respectively (detailed in the Supplementary Methods). In validation cohort, the endpoint of the study was defined as MACCEs (including all-cause death, AMI, stroke and TVR), stroke and cardiac events. The follow-up data were obtained through outpatient clinic visits, telephone interviews, or readmission, and the endpoints were adjudicated by an independent committee.

### Ascertainment of covariates

Information on demographics, geographic location, medical history, lifestyle habits, educational level, marital status and physical measurements was collected by trained interviewers in accordance with standard procedures (detailed in the Supplementary Methods). The covariates in the validation cohort have been described by previous studies (29).

### Statistical analyses

RStudio 4.2.1 software was employed for all statistical analyses. Two-sided *P* values < 0.05 were considered statistically significant. The normal distribution and equality of variance of continuous variables were assessed using the Kolmogorov–Smirnov test and Levene test, respectively. Continuous datasets were described using mean ± standard deviation (SD) or median (interquartile range) and compared using one-way analysis of variance or a nonparametric test, as appropriate. Nominal variables were presented as counts and percentages, and differences were identified using the chi-square test.

Kaplan–Meier curves were used to estimate the cumulative incidence of events. The association of both TyG-BMI index and hsCRP with events was examined using Cox models. Three models were established with incremental degrees of adjustment for potential confounders of events. Model 1 was a crude (unadjusted) model; Model 2 was adjusted for age, sex, systolic blood pressure (SBP), and diastolic blood pressure (DBP); Model 3 was adjusted for all variables included in Model 2 plus marital status, education, living place, serum creatinine, hemoglobin, uric acid (UA), BUN, TC, low-density lipoprotein cholesterol (LDL-C), hypertension, dyslipidemia, DM, smoking status, and alcohol consumption status. Schoenfeld residuals against time were examined to assess the proportional hazards assumption of these models.

Collinearity among the four groups, based on the discordant/concordant TyG-BMI and hsCRP, and other variables, was examined by computing the generalized variance inflation factor (GVIF). The GVIF^(1/2Df) of all included variables in our models was less than 2 (Supplementary File S1, Supplementary Tables S1–S3), indicating the absence of significant multicollinearity. Restricted cubic spline (RCS) curves were employed to examine the dose-relationship of both TyG-BMI index and hsCRP with CVD, with the multivariate adjustment mentioned above. The distribution of missing data for the included participants is presented in Supplementary File S1, Supplementary Table S4, and the multiple imputation method (random forest) was applied to impute the missing data.

## Results

### Characteristics of study participants

The baseline characteristics of enrolled participants, stratified by the median of TyG-BMI index, are presented in Table 1. Among the 6,534 middle-aged and elderly Chinese individuals included, the average age was 58.33 ± 8.80 years, and female patients accounted for 54.0% (*n* = 3,529). Participants with a high TyG-BMI index were younger, tended to be female, and exhibited higher levels of BP, heart rate, BMI, hemoglobin, FBG, HbA1c, TC, TG, LDL, and UA, while having a lower level of HDL, compared with those with a low TyG-BMI index. Additionally, a higher prevalence of hypertension, DM, dyslipidemia, and a higher proportion of living in the North areas and urban residence were observed in participants with a high TyG-BMI index. No statistically significant differences were found in the level of serum creatinine and the prevalence of kidney disease (all *P* values > 0.05).

**Table 1:** Baseline characteristics of participants by TyG-BMI.

Characteristics	Overall (*n* = 6,534)	Low TyG-BMI (*n* = 3,267)	High TyG-BMI (*n* = 3,267)	*P* value
Age, years	58.33 ± 8.80	59.44 ± 9.03	57.22 ± 8.41	<0.001
Female, *n* (%)	3,529 (54.0)	1,561 (47.8)	1,968 (60.2)	<0.001
SBP^b^, mmHg	128.37 ± 20.75	124.75 ± 20.17	132.00 ± 20.69	<0.001
DBP^b^, mmHg	74.95 ± 12.03	72.33 ± 11.61	77.58 ± 11.87	<0.001
Heart rate^b^, rpm	72.05 ± 10.20	71.02 ± 10.15	73.07 ± 10.15	<0.001
BMI, kg/m^2^	23.37 ± 3.53	20.78 ± 2.01	25.96 ± 2.73	<0.001
Rural residence, *n* (%)	4,417 (67.6)	2,408 (73.7)	2,009 (61.5)	<0.001
Region^a^, *n* (%)				<0.001
North	2,831 (43.3)	1,197 (36.6)	1,634 (50.0)	
South	3,703 (56.7)	2,070 (63.4)	1,633 (50.0)	
Education, *n* (%)				0.007
Junior high school and below	5,928 (90.7)	3,000 (91.8)	2,928 (89.6)	
Senior high school	552 (8.4)	241 (7.4)	311 (9.5)	
Tertiary	54 (0.8)	26 (0.8)	28 (0.9)	
Marital status, *n* (%)				<0.001
Married and living with spouse	5,571 (85.3)	2,713 (83.0)	2,858 (87.5)	
Others	963 (14.7)	554 (17.0)	409 (12.5)	
Alcohol consumption, *n* (%)	2,729 (41.8)	1,448 (44.3)	1,281 (39.2)	<0.001
Smoking, *n* (%)	2,521 (38.6)	1,464 (44.8)	1,057 (32.4)	<0.001
Hemoglobin^b^, g/dL	14.36 ± 2.20	14.19 ± 2.21	14.53 ± 2.16	<0.001
FBG, mg/dL	109.37 ± 35.00	101.67 ± 22.47	117.07 ± 42.75	<0.001
HbA1c^b^, %	5.25 ± 0.79	5.13 ± 0.57	5.38 ± 0.94	<0.001
hsCRP, mg/L	0.98 (0.53–2.06)	0.77 (0.45–1.69)	1.21 (0.66–2.35)	<0.001
TC, mg/dL	193.96 ± 38.62	187.62 ± 36.00	200.30 ± 40.09	<0.001
TG, mg/dL	132.71 ± 112.56	91.03 ± 43.28	174.39 ± 141.40	<0.001
HDL, mg/dL	51.56 ± 15.30	57.48 ± 15.21	45.64 ± 12.92	<0.001
LDL^b^, mg/dL	116.62 ± 34.74	113.83 ± 32.05	119.42 ± 37.04	<0.001
BUN^b^, mg/dL	15.71 ± 4.42	16.03 ± 4.62	15.38 ± 4.19	<0.001
UA, mg/dL	4.41 ± 1.22	4.26 ± 1.16	4.55 ± 1.25	<0.001
Serum creatinine^b^, mg/dL	0.77 ± 0.19	0.77 ± 0.19	0.77 ± 0.18	0.457
Hypertension, *n* (%)	2,474 (37.9)	920 (28.2)	1,554 (47.6)	<0.001
Kidney disease^b^, *n* (%)	313 (4.8)	165 (5.1)	148 (4.6)	0.318
Dyslipidemia, *n* (%)	459 (7.2)	101 (3.1)	358 (11.2)	<0.001
DM^b^, *n* (%)	315 (4.9)	69 (2.1)	246 (7.6)	<0.001

BMI, body mass index; BUN, blood urea nitrogen; DBP, diastolic blood pressure; DM, diabetes mellitus; FBG, fasting blood glucose; HbA1c, glycosylated hemoglobin A1c; HDL, high density lipoprotein; LDL, low density lipoprotein; SBP, systolic blood pressure; TC, total cholesterol; TG, triglycerides; TyG-BMI, triglyceride glucose-body mass index; UA, uric acid.

a Region was divided into north and south based on the Qinling Mountains-Huaihe River Line.

b data for some participants were missing.

The study population was further classified into the high-hsCRP group and low-hsCRP group, as summarized in Table 2. Compared with participants with low hsCRP, those with high hsCRP were older and had higher levels of BP, heart rate, BMI, UA, serum creatinine, FBG, HbA1c, and a higher prevalence of hypertension, DM, and dyslipidemia. The baseline characteristics of included participants stratified by the four groups (discordant/concordant TyG-BMI index and hsCRP) and CVD were presented in Supplementary File S1, Supplementary Tables S5, S6, respectively.

**Table 2:** Baseline characteristics of participants by hsCRP.

Characteristics	Overall (*n* = 6,534)	Low hsCRP (*n* = 4,862)	High hsCRP (*n* = 1,672)	*P* value
Age, years	58.33 ± 8.80	57.95 ± 8.69	59.42 ± 9.01	<0.001
Female, *n* (%)	3,529 (54.0)	2,641 (54.3)	888 (53.1)	0.392
SBP^b^, mmHg	128.37 ± 20.75	127.29 ± 20.38	131.54 ± 21.50	<0.001
DBP^b^, mmHg	74.95 ± 12.03	74.49 ± 11.99	76.31 ± 12.04	<0.001
Heart rate^b^, rpm	72.05 ± 10.20	71.64 ± 10.08	73.22 ± 10.45	<0.001
BMI, kg/m^2^	23.37 ± 3.53	23.10 ± 3.35	24.15 ± 3.92	<0.001
Rural residence, *n* (%)	4,417 (67.6)	3,339 (68.7)	1,078 (64.5)	0.002
Region^a^, *n* (%)				0.037
North	2,831 (43.3)	2,143 (44.1)	688 (41.1)	
South	3,703 (56.7)	2,719 (55.9)	984 (58.9)	
Education, *n* (%)				0.864
Junior high school and below	5,928 (90.7)	4,415 (90.8)	1,513 (90.5)	
Senior high school	552 (8.4)	406 (8.4)	146 (8.7)	
Tertiary	54 (0.8)	41 (0.8)	13 (0.8)	
Marital status, *n* (%)				0.009
Married and living with spouse	5,571 (85.3)	4,178 (85.9)	1,393 (83.3)	
Others	963 (14.7)	684 (14.1)	279 (16.7)	
Alcohol consumption, *n* (%)	2,729 (41.8)	2,049 (42.1)	680 (40.7)	0.292
Smoking, *n* (%)	2,521 (38.6)	1,825 (37.5)	696 (41.6)	0.003
Hemoglobin^b^, g/dL	14.36 ± 2.20	14.33 ± 2.16	14.47 ± 2.30	0.020
FBG, mg/dL	109.37 ± 35.00	107.57 ± 30.57	114.59 ± 45.11	<0.001
HbA1c^b^, %	5.25 ± 0.79	5.21 ± 0.73	5.37 ± 0.93	<0.001
hsCRP, mg/L	0.98 (0.53–2.06)	0.72 (0.45–1.14)	3.61 (2.60–6.28)	<0.001
TC, mg/dL	193.96 ± 38.62	193.84 ± 37.82	194.31 ± 40.87	0.668
TG, mg/dL	132.71 ± 112.56	128.75 ± 110.73	144.24 ± 116.99	<0.001
HDL, mg/dL	51.56 ± 15.30	52.65 ± 15.32	48.39 ± 14.79	<0.001
LDL^b^, mg/dL	116.62 ± 34.74	116.62 ± 33.88	116.60 ± 37.14	0.984
BUN^b^, mg/dL	15.71 ± 4.42	15.77 ± 4.38	15.54 ± 4.55	0.068
UA, mg/dL	4.41 ± 1.22	4.32 ± 1.19	4.67 ± 1.26	<0.001
Serum creatinine^b^, mg/dL	0.77 ± 0.19	0.77 ± 0.18	0.79 ± 0.20	<0.001
Hypertension, *n* (%)	2,474 (37.9)	1,710 (35.2)	764 (45.7)	<0.001
Kidney disease^b^, *n* (%)	313 (4.8)	236 (4.9)	77 (4.6)	0.667
Dyslipidemia, *n* (%)	459 (7.2)	310 (6.5)	149 (9.1)	<0.001
DM^b^, *n* (%)	315 (4.9)	208 (4.3)	107 (6.5)	<0.001

BMI, body mass index; BUN, blood urea nitrogen; DBP, diastolic blood pressure; DM, diabetes mellitus; FBG, fasting blood glucose; HbA1c, glycosylated hemoglobin A1c; HDL, high density lipoprotein; hsCRP, hypersensitive C-reactive protein; LDL, low density lipoprotein; SBP, systolic blood pressure; TC, total cholesterol; TG, triglycerides; TyG-BMI, triglyceride glucose-body mass index; UA, uric acid.

a Region was divided into north and south based on the Qinling Mountains-Huaihe River Line.

b Data for some participants were missing.

In the validation cohort, the baseline characteristics of 805 patients underwent PCI, stratified by the median of TyG-BMI index, are presented in Supplementary Table S6. Among the 805 patients underwent PCI included, the average age was 68.25 ± 6.15 years, and male patients accounted for 60.2% (*n* = 485). Participants with a high TyG-BMI index were younger, tended to be female, and exhibited higher levels of BMI, FBG, HbA1c, TC, TG, and LDL, while having a lower level of HDL and UA, compared with those with a low TyG-BMI index (Supplementary File S1, Supplementary Table S7).

### Association of TyG-BMI index and hsCRP with outcomes

**Figure 2:**
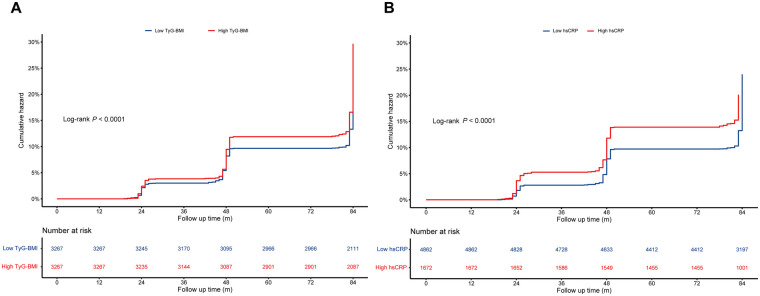
Kaplan–Meier curves for the cumulative incidence of CVD according to the TyG-BMI index **(A)** and hsCRP **(B)**. CVD, cardiovascular disease; hsCRP, high-sensitivity C-reactive protein; TyG-BMI, triglyceride glucose-body mass.

During a median follow-up of 84.0 months, 1,488 CVD events were documented, including 1,135 cardiac events and 486 strokes. Kaplan–Meier survival curves showed that participants with a high TyG-BMI index had a higher cumulative incidence of CVD (Figure 2A), stroke (Supplementary File S2, Supplementary Figure S1A), and cardiac events (Supplementary File S3, Supplementary Figure S2A), compared to those with a low TyG-BMI index. Similarly, cumulative rates of CVD (Figure 2B), stroke (Supplementary File S2, Supplementary Figure S1B), and cardiac events (Supplementary File S3, Supplementary Figure S2B) were much higher among individuals with a high hsCRP.

**Figure 3:**
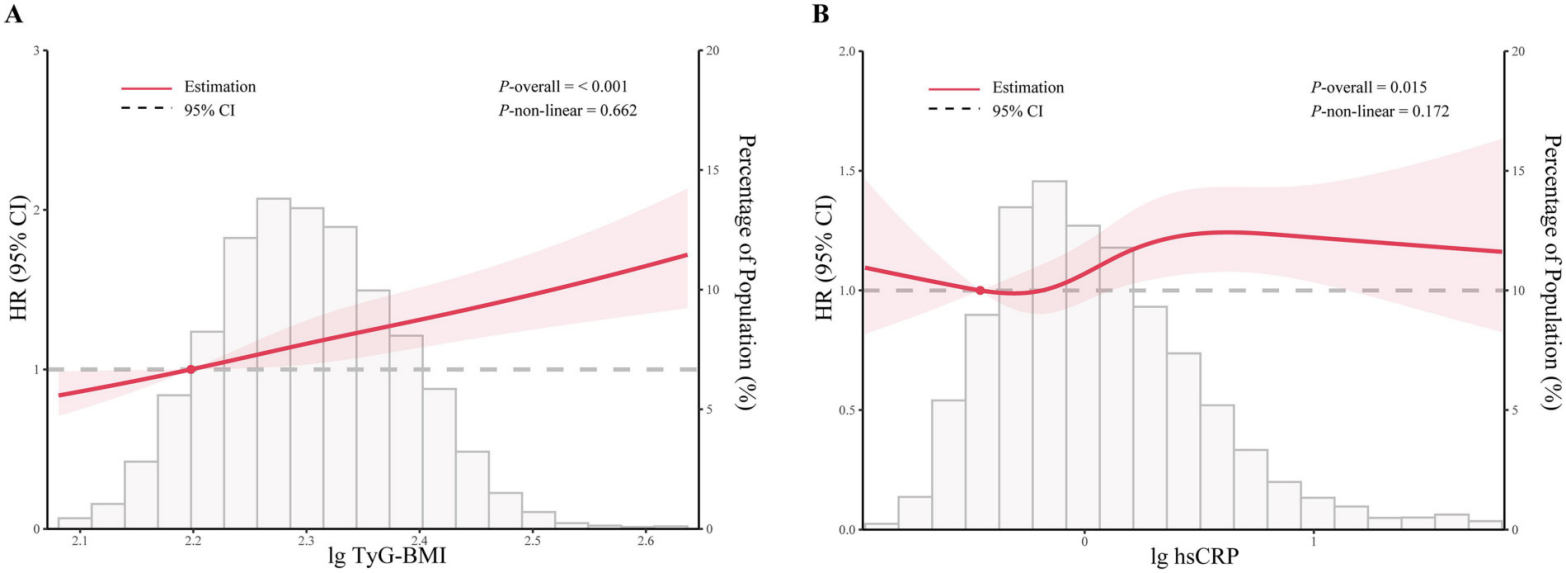
Restricted cubic spline curves for CVD according to the TyG-BMI index **(A)** and hsCRP **(B)** lg TyG-BMI, log-transformed TyG-BMI; lg hsCRP, log-transformed hsCRP. Hazard ratios are indicated by solid lines and 95% CIs by shaded areas. The horizontal dotted line represents the hazard ratio of 1.0. The model is adjusted for age, sex, SBP, DBP, marital status, education, living place, serum creatine, hemoglobin, uric acid, BUN, TC, LDL-C, hypertension, dyslipidemia, DM, smoking status and alcohol consumption status.

As shown in Table 3, the HR with 95% CI for participants with a high TyG-BMI index regarding CVD was 1.29 (95% CI: 1.17–1.43) in Model 1 (crude model). Although the association was slightly weakened, it remained significant in Model 2 (HR: 1.24, 95% CI: 1.12–1.39) and Model 3 (HR: 1.15, 95% CI: 1.03–1.30). A similar association of TyG-BMI index with either stroke or cardiac events was also observed. Regarding the association of hsCRP with outcomes (Table 4), after fully adjusting for potential covariates, a significant association of high hsCRP with CVD (HR: 1.21, 95% CI: 1.07–1.36), stroke (HR: 1.35, 95% CI: 1.11–1.64), and cardiac events (HR: 1.17, 95% CI: 1.02–1.34).In addition, as shown in Figure 3, RCS curves showed a significant dose-response relationship of either TyG-BMI index or hsCRP with CVD.

**Table 3:** Association of TyG-BMI with new-onset CVD, stroke and cardiac events.

Variables	Model 1		Model 2		Model 3	
	HR (95% CI)	*P* value	HR (95% CI)	*P* value	HR (95% CI)	*P* value
CVD						
Low TyG-BMI	Ref		Ref		Ref	
High TyG-BMI	1.29 (1.17–1.43)	<0.001	1.24 (1.12–1.39)	<0.001	1.15 (1.03–1.30)	0.016
Stroke						
Low TyG-BMI	Ref		Ref		Ref	
High TyG-BMI	1.48 (1.24–1.78)	<0.001	1.38 (1.14–1.67)	0.001	1.25 (1.02–1.54)	0.033
Cardiac events						
Low TyG-BMI	Ref		Ref		Ref	
High TyG-BMI	1.27 (1.13–1.43)	<0.001	1.24 (1.10–1.40)	0.001	1.15 (1.01–1.31)	0.042

Model 1: unadjusted.

Model 2: adjusted for age, sex, SBP and DBP.

Model 3: model 2 + further adjusted for marital status, education, living place, serum creatinine, hemoglobin, uric acid, BUN, TC, LDL-C, hypertension, dyslipidemia, DM, smoking status and alcohol consumption status.

**Table 4:** Association of hsCRP with new-onset CVD, stroke and cardiac events.

Variables	Model 1		Model 2		Model 3	
	HR (95% CI)	*P* value	HR (95% CI)	*P* value	HR (95% CI)	*P* value
CVD						
Low hsCRP	Ref		Ref		Ref	
High hsCRP	1.32 (1.18–1.48)	<0.001	1.25 (1.12–1.39)	<0.001	1.21 (1.07–1.36)	0.001
Stroke						
Low hsCRP	Ref		Ref		Ref	
High hsCRP	1.56 (1.29–1.89)	<0.001	1.42 (1.18–1.72)	<0.001	1.35 (1.11–1.64)	0.003
Cardiac events						
Low hsCRP	Ref		Ref		Ref	
High hsCRP	1.26 (1.11–1.43)	<0.001	1.20 (1.06–1.37)	0.005	1.17 (1.02–1.34)	0.023

Model 1: unadjusted.

Model 2: adjusted for age, sex, SBP and DBP.

Model 3: model 2 + further adjusted for marital status, education, living place, serum creatinine, hemoglobin, uric acid, BUN, TC, LDL-C, hypertension, dyslipidemia, DM, smoking status and alcohol consumption status.

**Figure 4:**
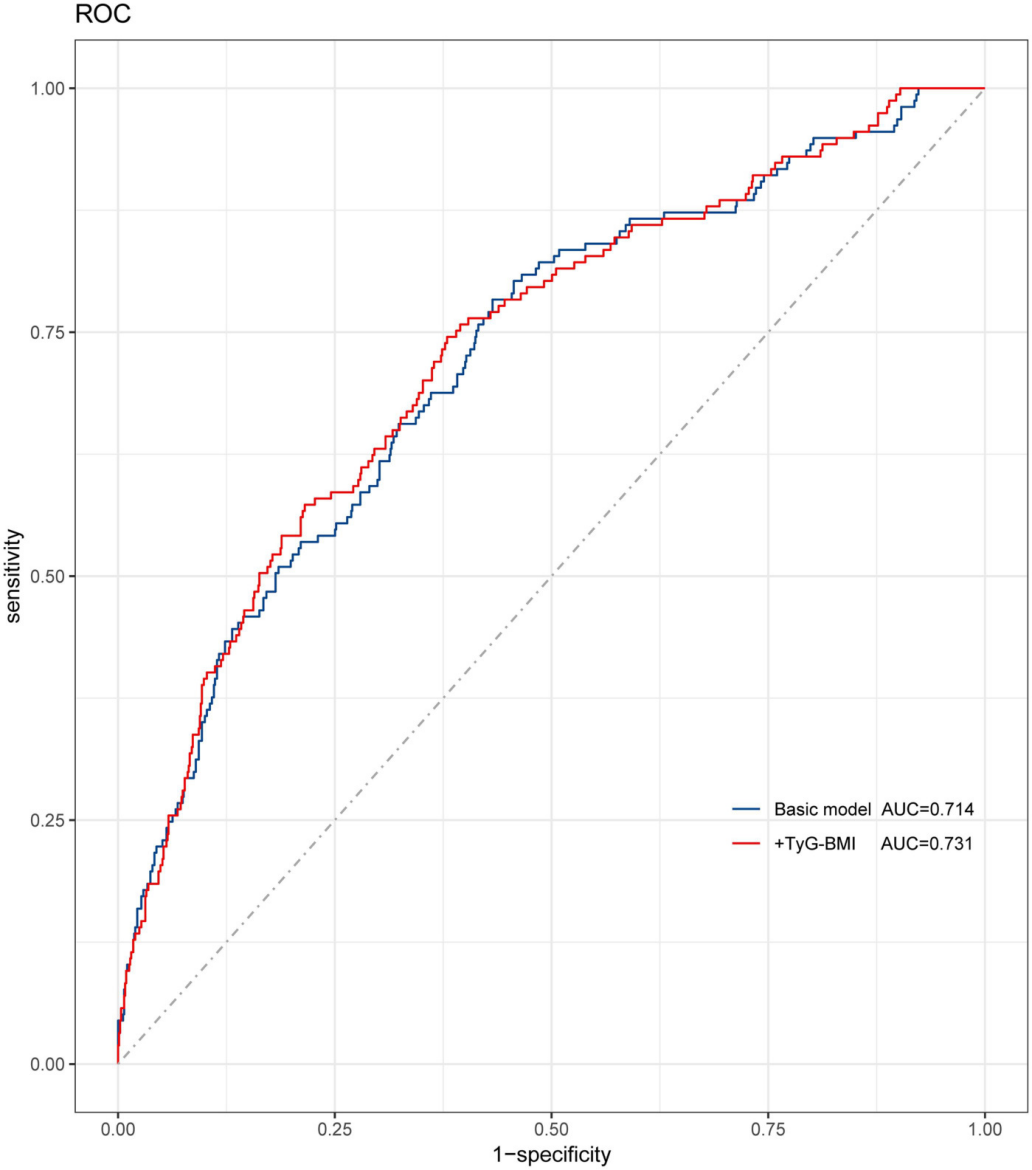
The receiver operating characteristic curves of the TyG-BMI index as a marker to predict MACCEs among patients underwent PCI. Basic risk model vs.+the TyG-BMI index. Basic risk model includes age, heart failure, previous AMI, creatinine, uric acid, ACEI, number of diseased vessels, LAD, RCA, CTO, and length of stents.

The validation cohort included a total of 805 patients receiving at least one DES at baseline. During the 34-month followup period, 137 cases (17.0%) experienced incident MACCEs. A high TyG-BMI index had a higher risk of MACCEs, stroke, and cardiac events, compared to those with a low TyG-BMI index (Table 5). Additionally, the predictive performance of the model was significantly improved after adding TyG-BMI to the baseline model (Figure 4).

**Table 5:** Association of TyG-BMI with new-onset MACCEs, stroke and cardiac events among patients underwent PCI.

Variables	Model 1		Model 2		Model 3	
	OR (95% CI)	*P* value	OR (95% CI)	*P* value	OR (95% CI)	*P* value
MACCEs						
Low TyG-BMI	Ref		Ref		Ref	
High TyG-BMI	1.24 (1.05–1.47)	0.021	1.28 (1.09–1.52)	0.010	1.26 (1.07–1.51)	0.016
Stroke						
Low TyG-BMI	Ref		Ref		Ref	
High TyG-BMI	1.12 (0.97–1.32)	0.120	1.18 (0.99–1.40)	0.061	1.25 (1.02–1.54)	0.033
Cardiac events						
Low TyG-BMI	Ref		Ref		Ref	
High TyG-BMI	1.27 (1.13–1.43)	0.024	1.31 (1.10–1.56)	0.008	1.25 (1.06–1.49)	0.017

CI, confidence interval; OR, odds ratio.

Model 1: unadjusted.

Model 2: adjusted for age, hypertension, and diabetes mellitus.

Model 3: model 2 + further adjusted for heart failure, previous AMI, creatinine, uric acid, ACEI, number of diseased vessels, LAD, RCA, length of stents, and diameters of stents.

### Joint effect of the TyG-BMI index and hsCRP on outcomes

**Figure 5:**
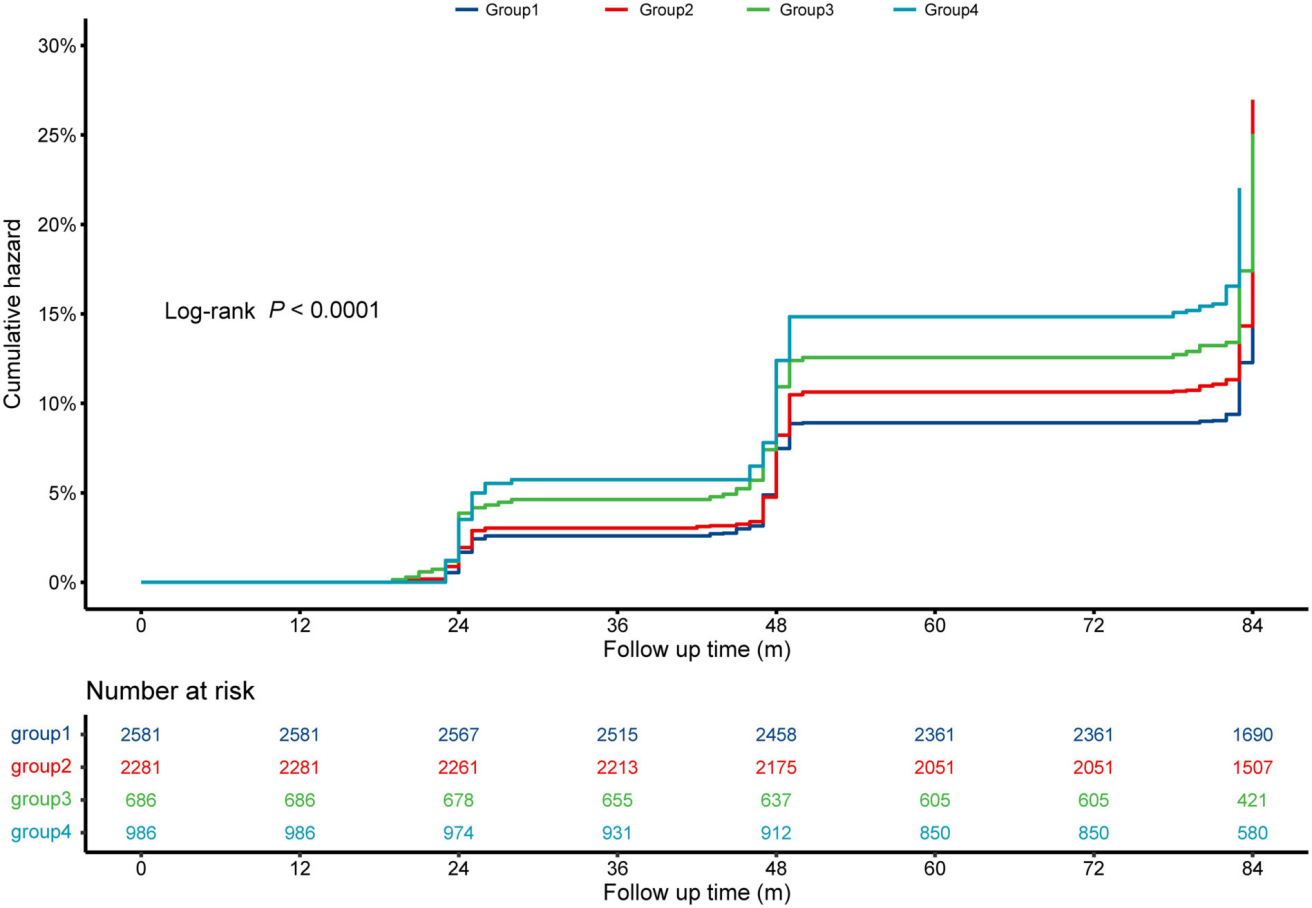
Kaplan–Meier curves for the cumulative incidence of CVD across TyG-BMI index and hsCRP groups. Group 1 is low TyG-BMI and low hsCRP; Group 2 is high TyG-BMI and low hsCRP; Group 3 is low TyG-BMI and high hsCRP; Group 4 is high TyG-BMI and high hsCRP. CVD, cardiovascular disease; hsCRP, high-sensitivity C-reactive protein; TyG-BMI, triglyceride glucose-body mass.

Participants were grouped into four groups based on discordant/concordant TyG-BMI index and hsCRP. The results indicated that participants in Group 4 (high TyG-BMI and high hsCRP) had the highest risk of CVD (Figure 5), stroke (Supplementary File S2, Supplementary Figure S1C), and cardiac events (Supplementary File S3, Supplementary Figure S2C), while those in Group 1 (low TyG-BMI and low hsCRP) had the lowest cumulative incidence of these events (all log-rank test, *P* < 0.0001).

The combined influences of TyG-BMI index and hsCRP on new-onset CVD, stroke, and cardiac events were further evaluated (Table 6). As expected, the highest risk of CVD was observed among participants in Group 4 (high TyG-BMI and high hsCRP) (HR: 1.65, 95% CI: 1.42–1.90). The correlation remained significant even in Model 3 (HR: 1.38, 95% CI: 1.18–1.62). And the highest risk of incident stroke (HR: 1.61, 95% CI: 1.21–2.14), and cardiac events (HR: 1.36, 95% CI: 1.13–1.63), was also found among participants in Group 4.

**Table 6:** Joint association of TyG-BMI and hsCRP with new-onset CVD, stroke and cardiac events.

Variables	Model 1		Model 2		Model 3	
	HR (95% CI)	*P* value	HR (95% CI)	*P* value	HR (95% CI)	*P* value
CVD						
Group 1	Ref		Ref		Ref	
Group 2	1.23 (1.09–1.39)	0.001	1.18 (1.04–1.34)	0.010	1.11 (0.97–1.27)	0.125
Group 3	1.21 (1.01–1.45)	0.043	1.13 (0.95–1.36)	0.174	1.12 (0.93–1.35)	0.225
Group 4	1.65 (1.42–1.90)	<0.001	1.51 (1.30–1.75)	<0.001	1.38 (1.18–1.62)	<0.001
Stroke						
Group 1	Ref		Ref		Ref	
Group 2	1.59 (1.28–1.99)	<0.001	1.46 (1.16–1.83)	0.001	1.35 (1.06–1.72)	0.015
Group 3	1.86 (1.37–2.51)	<0.001	1.62 (1.19–2.19)	0.002	1.60 (1.18–2.18)	0.003
Group 4	2.10 (1.62–2.73)	<0.001	1.83 (1.41–2.39)	<0.001	1.61 (1.21–2.14)	0.001
Cardiac events						
Group 1	Ref		Ref		Ref	
Group 2	1.16 (1.01–1.33)	0.035	1.14 (0.99–1.32)	0.073	1.07 (0.92–1.25)	0.379
Group 3	1.04 (0.84–1.29)	0.706	1.01 (0.81–1.25)	0.932	1.00 (0.80–1.54)	0.980
Group 4	1.57 (1.34–1.85)	<0.001	1.48 (1.25–1.75)	<0.001	1.36 (1.13–1.63)	0.001

Group 1 is low TyG-BMI and low hsCRP; Group 2 is high TyG-BMI and low hsCRP; Group 3 is low TyG-BMI and high hsCRP; Group 4 is high TyG-BMI and high hsCRP.

Model 1: unadjusted.

Model 2: adjusted for age, sex, SBP and DBP.

Model 3: model 2 + further adjusted for marital status, education, living place, serum creatinine, hemoglobin, uric acid, BUN, TC, LDL-C, hypertension, dyslipidemia, DM, smoking status and alcohol consumption status.

### Subgroup analyses

**Figure 6:**
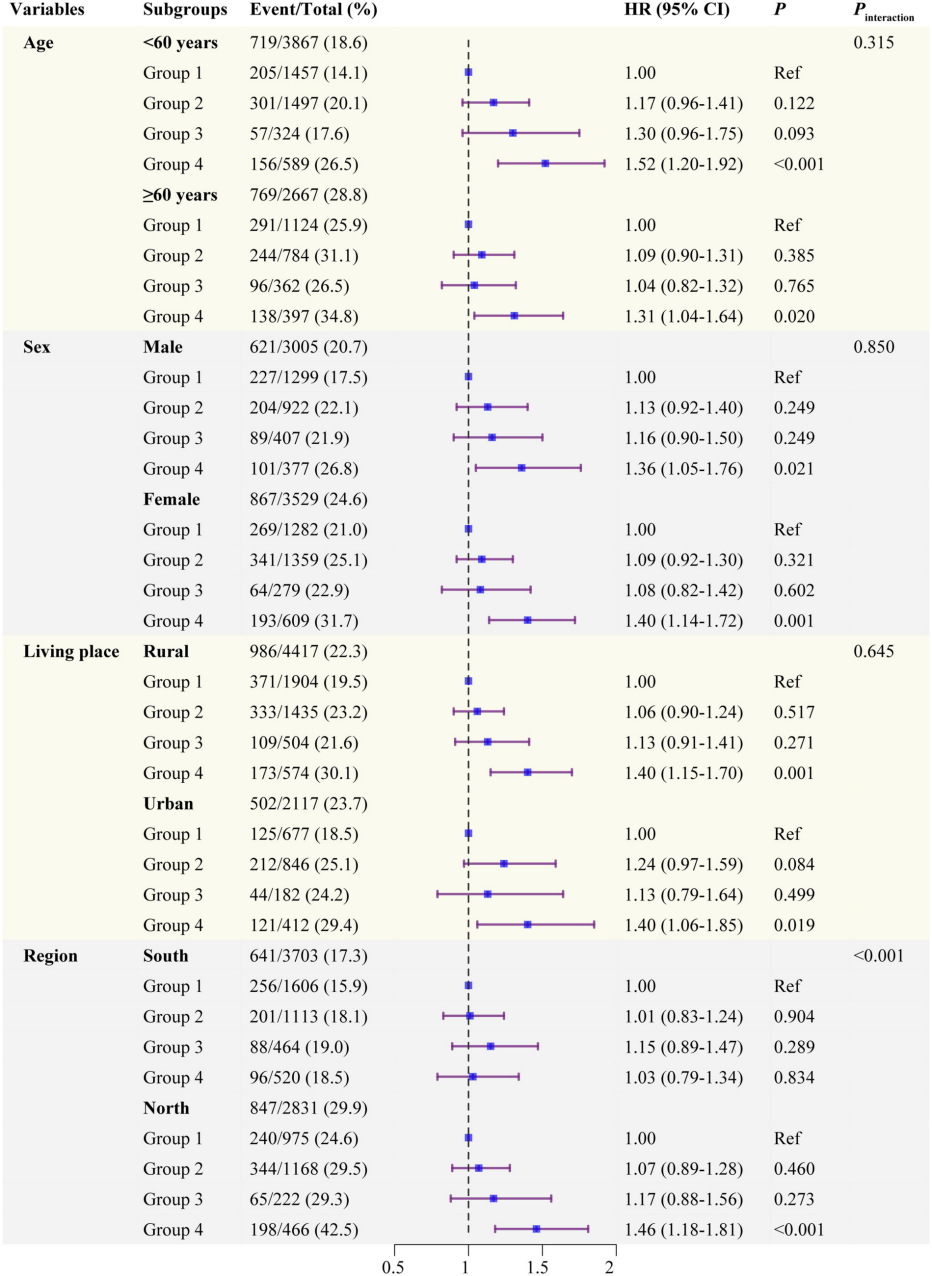
Subgroup and interaction analyses between the four groups and CVD across various subgroups. Group 1 is low TyG-BMI and low hsCRP; Group 2 is high TyG-BMI and low hsCRP; Group 3 is low TyG-BMI and high hsCRP; Group 4 is high TyG-BMI and high hsCRP.

Stratified analyses were further performed according to potential CVD risk factors. As shown in Figure 6, we found a significant interaction between the region subgroups and the impact of four groups on the incidence of CVD (*P* for interaction < 0.001): individuals living in North areas in Group 4 had a significantly elevated risk of CVD (HR: 1.46, 95% CI: 1.18–1.81). The other variables, including age, sex, residence, did not significantly modify the association. Similar results were also found in subgroup analyses for cardiac events (Supplementary File S4, Supplementary Figure S3). As presented in Supplementary File S5, Supplementary Figure S4, no significant interactions were observed among the subgroup analyses for stroke (all *P* for interaction > 0.05).

### Sensitivity analyses

In the sensitivity analyses, the combined association of TyG-BMI and hsCRP with CVD, stroke, and cardiac events in the adjusted full model were not appreciably changed when individuals who experienced CVD during or before Survey 2 were excluded (Supplementary File S1, Supplementary Table S8). After excluding individuals with extremely high TyG-BMI or hsCRP (>99% percentile), the fully adjusted model showed similar results (Supplementary File S1, Supplementary Table S9). Furthermore, when we excluded individuals with DM, the results remained largely unchanged (Supplementary File S1, Supplementary Table S10).

### Additional analyses

As shown in Supplementary File S6, Supplementary Figure S5, E-values for the combined influences of TyG-BMI index and hsCRP on cardiac events, stroke, and CVD were 2.06, 2.60, and 2.10, respectively, which indicated the correlations were not neutralized by the potential unmeasured confounding in the current study. In the imputed dataset, the joint association of TyG-BMI index and hsCRP with CVD, stroke, and cardiac events was consistent with the main results (Supplementary File S1, Supplementary Table S11).

## Discussion

Among 6,534 middle-aged and elderly individuals from CHARLS with a median of 84 months of follow-up, the main findings of our study were as follows: (1) elevated TyG-BMI index and hsCRP were associated with a respective 1.15-fold and 1.21-fold increase in the risk of incident CVD; (2) both the TyG-BMI index and hsCRP were also independent predictors of stroke and cardiac events; (3) participants who had concurrently high TyG-BMI index and hsCRP had the higher risk of CVD, stroke, and cardiac events, compared with those with either or neither risk factor elevated. Therefore, a comprehensive view of multiple risk factors may be a wiser and more effective strategy for risk stratification and improving the management of CVD.

Recently, increasing evidence has suggested that IR plays an indispensable role in the pathogenesis of atherosclerosis and clinically relevant advanced plaque progression (9). IR is not only considered a major risk factor for CVD (10, 11), but it also predicts adverse cardiovascular events in patients with CVD (30). Therefore, there is an urgent need for the early identification of IR to improve the risk stratification of CVD. Although the hyperinsulinemic-euglycemic clamp is considered the gold-standard method for evaluating IR, it appears to be used mainly in academic studies due to its invasive nature and expensive cost. To suit clinical practice and large-scale epidemiological studies, the TyG index was proposed to assess IR and has been confirmed to have a good correlation with the hyperinsulinemic-euglycemic clamp in assessing IR (13, 14). As is well-known, obesity, usually reflected by BMI, has been regarded as a crucial factor in IR (15). Then, the TyG-BMI index, combining the TyG index with BMI, was found to have good agreement with the homeostasis model assessment for IR in assessing IR in the Chinese population without DM (17). Moreover, the superiority of the TyG-BMI index was further confirmed in the Korean population (16). A study showed that the TyG-BMI index had a linear association with ischemic stroke and could significantly improve the predictive power of the basic risk model in estimating the prevalence of ischemic stroke (31). Moreover, a recent study reported that an elevated TyG-BMI index was significantly associated with a higher incidence of adverse cardiovascular events in female patients or elderly patients undergoing percutaneous coronary intervention (32). All the above evidence suggests that the TyG-BMI index may serve as a promising predictor of CVD in the general population. In our prospective, longitudinal study, the TyG-BMI index also proved to be a reliable predictor for future CVD (HR: 1.15, 95% CI: 1.03–1.30, *P* = 0.016), stroke (HR: 1.25, 95% CI: 1.02–1.54, *P* = 0.033), and cardiac events (HR: 1.15, 95% CI: 1.01–1.31, *P* = 0.042), independently of traditional cardiovascular risk factors.

Furthermore, existing evidence indicates that low-grade chronic inflammation is implicated in the onset and progression of CVD (18, 19). And Patients with CVD exhibit a more pronounced inflammatory condition, as confirmed by detecting hsCRP (33, 34). To date, several randomized controlled trials have examined the association between inflammation and cardiovascular events (22, 23, 35). The JUPITER study demonstrated that rosuvastatin significantly reduced the incidence of major cardiovascular events by 37% through a reduction in hsCRP among participants without hyperlipidemia but with elevated levels of hsCRP (22). Subsequently, the recently published FOURIER study provided additional evidence supporting a positive association between elevated hsCRP and cardiac death as well as stroke (all *P* < 0.001) (23). The *post hoc* analysis of the SPIRE trials similarly yielded results, indicating that hsCRP > 3 mg/L was associated with a 60% higher risk of incident cardiovascular events (35). Importantly, the CANTOS study showed that canakinumab, an interleukin-1 (IL-1) inhibitor, significantly decreased hsCRP levels and the incidence of cardiovascular events in individuals with a history of myocardial infarction and elevated hsCRP (24). The effectiveness of reducing the risk of cardiovascular events with anti-inflammatory therapies has been well established (24, 36). Similarly, in the current study, an elevated hsCRP was linked to an increased risk of CVD (HR: 1.21, 95% CI: 1.07–1.36, *P* = 0.001), stroke (HR: 1.35, 95% CI: 1.11–1.64, *P* = 0.003), and cardiac events (HR: 1.17, 95% CI: 1.02–1.34, *P* = 0.023).

Recently, there has been an increasing focus on the importance of comprehensively evaluating and controlling multiple risk factors to stratify and reduce CVD risk. According to previous reports, the increased risk of cardiovascular events is often synergistic rather than additive in the presence of multiple risk factors (37, 38). Wu et al. reported that individuals with only TyG index > 8.87, only hyperuricemia, and both TyG index > 8.87 and hyperuricemia experienced respective increments of 111%, 67%, and 310% in the risk of major adverse cardiovascular events (38). As mentioned earlier, hsCRP may accelerate cardiovascular risk by contributing to insulin resistance (IR) (25, 26). Recognizing the close association between inflammation and insulin resistance (IR) with cardiovascular risk, and the existing knowledge gap regarding the synergistic effect of inflammation and IR in elevating the risk of incident CVD, stroke, and cardiac events, we designed the present study. To the best of our knowledge, we present the initial evidence demonstrating that elevated levels of both TyG-BMI index and hsCRP synergistically increase the risk of CVD, stroke, and cardiac events to the greatest extent.

While the exact mechanisms underlying the association of elevated TyG-BMI index and hsCRP with CVD, stroke, and cardiac events remain unclear, they may be explained at least partly as follows: (1) Elevated hsCRP may facilitate IR via the excessive release of proinflammatory cytokines, such as IL-1β, activating the innate immune system (39); (2) High circulating insulin concentrations could reduce the production of nitric oxide by activating serum and glucocorticoid kinase 1. The lower nitric oxide concentration, in turn, leads to matrix protein deposition and fibrosis (40); (3) The metabolic disorders of serum lipids and glucose induced by IR may cause the overproduction of reactive oxygen species, which may contribute to cardiovascular events (41); (4) Both inflammation and IR promote thrombosis and platelet aggregation in the cardiovascular system by impairing fibrinolysis (19, 42).

Our study has several strengths. Firstly, CHARLS is a well-established population-based prospective cohort with a relatively large sample size and a nationally representative sample, which enhances the generalizability of our findings. Secondly, we had abundant information on demographics, geographic location, and medical history, allowing us to fully adjust for potential confounders. Finally, the measurements of lipid parameters, hsCRP, and BMI were based on standard reliable methods, ensuring the confidence of our results. However, our study is not without limitations. Firstly, potential reverse causality may exist. However, even after excluding participants who experienced stroke or cardiac events during or before Survey 2, the results remained consistent with the main results. Secondly, although we adjusted for many potential confounders, residual or unmeasured confounding was unavoidable. Importantly, E-value analyses suggested that the associations were not neutralized by potential unmeasured confounding. Thirdly, stroke and cardiac events were defined using self-reported physician diagnoses, which may introduce information bias. Therefore, large-scale randomized controlled trials are needed to further examine these findings. Lastly, our study only included participants aged 45 years and older, limiting the generalizability of our results to the general population.

## Conclusions

In conclusion, the results from the present study, for the first time, reveal synergistic relationships between the TyG-BMI index and hsCRP in predicting the risk of CVD, stroke, and cardiac events. Importantly, TyG-BMI also demonstrated significant predictive value for adverse outcomes in patients after PCI. These novel findings underscore the urgent need for using multiple assessments concurrently in risk stratification, which could have far-reaching significance for the primary or secondary prevention of CVD and improving public health.

## Data Availability

Publicly available datasets were analyzed in this study. This data can be found here: http://charls.pku.edu.cn/en.
