# Supplementary material for: Combining triglyceride glucose-body mass index and high-sensitivity C-reactive protein to predict cardiovascular disease: results from a national cohort and a clinical verified cohort
Source: Front Cardiovasc Med. 2026 Jan 7;12:1726615. doi: 10.3389/fcvm.2025.1726615 (PMC12819627; doi:10.3389/fcvm.2025.1726615)
Supplement: Supplementary file 1 [file Datasheet1.docx]

**Combining triglyceride glucose-body mass index and high-sensitivity C-reactive protein to predict cardiovascular disease: Results from a national cohort and a clinical verified cohort**

**Supplementary Methods**

## Section 1. Detailed information about sampling selection

In brief, a four-stage, stratified, clustering sample method was employed to obtain a nationally representative sample. The baseline survey of CHARLS took place between 2011 and 2012 (wave 1), with a total enrollment of 17,708 residents from 28 provinces on the Chinese mainland.[1]. Four subsequent follow-ups were completed in 2013 (wave 2), 2015 (wave 3), 2018 (wave 4), and 2020 (wave 5), respectively. However, the 2020 data have not been released to the public at the time of writing.

## Section 2. Ascertainment of exposure

After an overnight fast, blood samples of CHARLS participants at baseline were collected by professional staff, stored at − 20℃, and transported to Beijing. Further measurements were conducted following standard procedures. FBG and TG were measured using enzymatic colorimetric assays. A digital weighing scale (Omron Corporation, HN‐286) and a stadiometer (Seca Corporation, 213) were utilized to measure body mass and height with precision to 0.1 kg and 0.1 cm, respectively. Measurements were taken while individuals wore lightweight clothes and no shoes.

## Section 3. Outcome assessment

Detailed information on the outcome was collected by a standardized questionnaire: ‘have you been diagnosed by a doctor with heart attack, coronary heart disease, or stroke?’, which is widely used and accepted in international leading aging surveys, such as the Health and Retirement Study (HRS) and the English Longitudinal Study of Aging (ELSA) and the Survey of Health[2]. To ensure data reliability, quality control of data recording and checking was implemented. Participants were followed from baseline (2011) to the onset of stroke and cardiac events or the most recent survey (2018), whichever occurred first.

## Section 4. Ascertainment of covariates

After at least 5 minutes of sitting rest, the BP of the participants was measured three times by a trained interviewer at 45-second intervals using a digital sphygmomanometer (Omron TM HEM-7200). Serum lipid parameters were measured using enzymatic colorimetric assays. Blood urea nitrogen (BUN) was measured using an enzymatic UV method involving urease. Serum concentrations of creatinine and uric acid, as well as glycosylated hemoglobin A1c (HbA1c), were measured following standard procedures.

## Section 5. Definitions

The Qinling Mountains-Huaihe River Line was used to delineate the North and South areas[3]. Participants with hypertension were defined based on a self-reported physician diagnosis (a positive response to “Have you been diagnosed with hypertension?”), and/or recent use of an antihypertensive agent (a positive response to “Are you currently taking any antihypertensive drugs to treat or control your BP?”), and/or a blood pressure (BP) reading ≥140/90 mmHg[4]. DM was defined based on a self-reported physician diagnosis (a positive response to “Have you been diagnosed with DM?”), use of insulin and hypoglycemic medication, or FBG ≥ 126 mg/dL, and/or an HbA1c level ≥ 6.5% at baseline[5]. Kidney disease was defined as self-reported physician diagnosis (a positive response to “Have you been diagnosed with any kidney disease [excluding tumor or cancer]?”), following the methodology used in the previous CHARLS study[6]. Dyslipidemia was diagnosed based on self-reported physician diagnosis (a positive response to “Have you been diagnosed with dyslipidemia?”), current use of any lipid-lowering drugs, and specific lipid levels: total cholesterol (TC) ≥ 240 mg/dl, TG ≥150 mg/dl, high-density lipoprotein (HDL) <40 mg/dl, LDL ≥160 mg/dl[6].

## Section 6. Subgroup and sensitivity analyses

Furthermore, subgroup analyses were conducted to examine if the relationship was modified by age (<60 or ≥60 years), sex (male or female), residence (rural or urban area), and region (South or North). To assess the robustness of the main results, several sensitivity analyses were performed. First, participants who developed CVD during or before Survey 2 were excluded to reduce the potential reverse causation bias. Second, individuals with extremely high TyG-BMI or hsCRP (>99th percentile) were further excluded. Third, participants with DM at baseline were excluded. Fourth, the analysis was repeated using the imputed dataset. Finally, to evaluate the extent of unmeasured confounding, E-value analyses, defined as the minimum strength of correlation, were performed based on the estimated hazard ratio (HR) and 95% confidence interval (CI) for events[7].

**Table S1.** Analysis of collinearity for cardiac events in model 3

| **Variables** | **GVIG** | **Df** | **GVIF^(1/(2*Df))** |
| --- | --- | --- | --- |
| Age | 1.454732 | 1 | 1.206123 |
| Gender | 2.794921 | 1 | 1.671802 |
| Education | 1.114705 | 2 | 1.027519 |
| Rural residence | 1.062683 | 1 | 1.030865 |
| DM | 1.113632 | 1 | 1.055288 |
| Dyslipidemia | 1.154097 | 1 | 1.074289 |
| SBP | 3.236723 | 1 | 1.799089 |
| DBP | 2.507193 | 1 | 1.583412 |
| BUN | 1.155565 | 1 | 1.074972 |
| Serum creatine | 1.716526 | 1 | 1.310163 |
| TC | 3.411502 | 1 | 1.847025 |
| LDL | 3.268535 | 1 | 1.807909 |
| UA | 1.549199 | 1 | 1.244668 |
| Hemoglobin | 1.163914 | 1 | 1.078848 |
| Marital status | 1.091372 | 1 | 1.044687 |
| Alcohol consumption | 1.433594 | 1 | 1.197328 |
| Smoking | 2.011418 | 1 | 1.418245 |
| Hypertension | 1.917577 | 1 | 1.384766 |
| Groups | 1.318171 | 3 | 1.047117 |

Dependent variable: Groups (Group 1 is low TyG-BMI and low hsCRP; Group 2 is high TyG-BMI and low hsCRP; Group 3 is low TyG-BMI and high hsCRP; Group 4 is high TyG-BMI and high hsCRP)

GVIF, generalized variance inflation factor; DF, degree of freedom. (GVIF^(1/(2*Df)) ≥ 2 indicates collinearity)

Collinearity analysis showed that no significant collinearity.

BUN, blood urea nitrogen; DBP, diastolic blood pressure; DM, diabetes mellitus; LDL, low density lipoprotein; SBP, systolic blood pressure; TC, total cholesterol; UA, uric acid

**Table S2.** Analysis of collinearity for stroke in model 3

| **Variables** | **GVIG** | **Df** | **GVIF^(1/(2*Df))** |
| --- | --- | --- | --- |
| Age | 1.46678257 | 1 | 1.211107993 |
| Gender | 2.884430993 | 1 | 1.698361267 |
| Education | 1.086600033 | 2 | 1.020980455 |
| Rural residence | 1.049947215 | 1 | 1.02466932 |
| DM | 1.094274376 | 1 | 1.046075703 |
| Dyslipidemia | 1.146310804 | 1 | 1.070659051 |
| SBP | 3.138454082 | 1 | 1.771568255 |
| DBP | 2.527114332 | 1 | 1.589690011 |
| BUN | 1.175763945 | 1 | 1.084326494 |
| Serum creatine | 1.731480054 | 1 | 1.315857156 |
| TC | 2.947125686 | 1 | 1.716719455 |
| LDL | 2.83600014 | 1 | 1.684042796 |
| UA | 1.562215294 | 1 | 1.249886112 |
| Hemoglobin | 1.181922523 | 1 | 1.087162602 |
| Marital status | 1.121912355 | 1 | 1.059203642 |
| Alcohol consumption | 1.487089188 | 1 | 1.219462664 |
| Smoking | 2.026396643 | 1 | 1.423515593 |
| Hypertension | 1.910505418 | 1 | 1.382210338 |
| Groups | 1.318963288 | 3 | 1.047222066 |

Dependent variable: Groups (Group 1 is low TyG-BMI and low hsCRP; Group 2 is high TyG-BMI and low hsCRP; Group 3 is low TyG-BMI and high hsCRP; Group 4 is high TyG-BMI and high hsCRP)

GVIF, generalized variance inflation factor; DF, degree of freedom. (GVIF^(1/(2*Df)) ≥ 2 indicates collinearity)

Collinearity analysis showed that no significant collinearity.

BUN, blood urea nitrogen; DBP, diastolic blood pressure; DM, diabetes mellitus; LDL, low density lipoprotein; SBP, systolic blood pressure; TC, total cholesterol; UA, uric acid

**Table S3.** Analysis of collinearity for CVD in model 3

| **Variables** | **GVIG** | **Df** | **GVIF^(1/(2*Df))** |
| --- | --- | --- | --- |
| Age | 1.457173986 | 1 | 1.207134618 |
| Gender | 2.829280571 | 1 | 1.682046543 |
| Education | 1.100863608 | 2 | 1.024314637 |
| Rural residence | 1.056885371 | 1 | 1.028049304 |
| DM | 1.107598711 | 1 | 1.052425157 |
| Dyslipidemia | 1.151459725 | 1 | 1.073060914 |
| SBP | 3.250456275 | 1 | 1.802902181 |
| DBP | 2.533054178 | 1 | 1.591557155 |
| BUN | 1.159969467 | 1 | 1.077018787 |
| Serum creatine | 1.716986826 | 1 | 1.310338439 |
| TC | 3.235154896 | 1 | 1.798653634 |
| LDL | 3.095566671 | 1 | 1.759422255 |
| UA | 1.550697119 | 1 | 1.245269898 |
| Hemoglobin | 1.168569473 | 1 | 1.081003919 |
| Marital status | 1.097340584 | 1 | 1.047540254 |
| Alcohol consumption | 1.455847343 | 1 | 1.206584992 |
| Smoking | 2.029189571 | 1 | 1.424496252 |
| Hypertension | 1.94078565 | 1 | 1.393120831 |
| Groups | 1.321576626 | 3 | 1.047567601 |

Dependent variable: Groups (Group 1 is low TyG-BMI and low hsCRP; Group 2 is high TyG-BMI and low hsCRP; Group 3 is low TyG-BMI and high hsCRP; Group 4 is high TyG-BMI and high hsCRP)

GVIF, generalized variance inflation factor; DF, degree of freedom. (GVIF^(1/(2*Df)) ≥ 2 indicates collinearity)

Collinearity analysis showed that no significant collinearity.

BUN, blood urea nitrogen; CVD, cardiovascular disease; DBP, diastolic blood pressure; DM, diabetes mellitus; LDL, low density lipoprotein; SBP, systolic blood pressure; TC, total cholesterol; UA, uric acid

**Table S4.** Distribution of missing data.

| **Characteristics** | **No. of missing values** | **Percent(%)** | **Disposition** |
| --- | --- | --- | --- |
| LDL | 12 | 0.18 | Multiple imputation |
| Serum creatinine | 3 | 0.05 | Multiple imputation |
| BUN | 1 | 0.02 | Multiple imputation |
| HbA1c | 45 | 0.69 | Multiple imputation |
| Hemoglobin | 110 | 1.68 | Multiple imputation |
| SBP | 51 | 0.78 | Multiple imputation |
| DBP | 52 | 0.80 | Multiple imputation |
| Heart rate | 53 | 0.81 | Multiple imputation |
| DM | 78 | 1.19 | Multiple imputation |
| Kidney disease | 47 | 0.72 | Multiple imputation |
| Total | 452 | 6.92 | Multiple imputation |

BUN, blood urea nitrogen; DBP, diastolic blood pressure; DM, diabetes mellitus; HbA1c, glycosylated hemoglobin A1c; LDL, low density lipoprotein; SBP, systolic blood pressure

**Table S5.** Baseline characteristics of enrolled participants among groups of discordant/concordant TyG-BMI and hsCRP

| **Characteristics** | **Overall**  **(n=6534)** | **Group 1**  **(n=2581)** | **Group 2**  **(n=2281)** | **Group 3**  **(n=686)** | **Group 4**  **(n=986)** | ***P* value** |
| --- | --- | --- | --- | --- | --- | --- |
| Age, years | 58.33 ± 8.80 | 59.02 ± 8.95 | 56.75 ± 8.22 | 61.01 ± 9.15 | 58.31 ± 8.74 | <0.001 |
| Female, n (%) | 3529 (54.0) | 1282 (49.7) | 1359 (59.6) | 279 (40.7) | 609 (61.8) | <0.001 |
| SBP^&^, mmHg | 128.37 ± 20.75 | 123.93 ± 19.66 | 131.09 ± 20.53 | 127.82 ± 21.76 | 134.14 ± 20.93 | <0.001 |
| DBP^&^, mmHg | 74.95 ± 12.03 | 72.00 ± 11.44 | 77.30 ± 11.98 | 73.55 ± 12.16 | 78.23 ± 11.58 | <0.001 |
| Heart rate^&^, rpm | 72.05 ± 10.20 | 70.75 ± 9.99 | 72.66 ± 10.09 | 72.06 ±10.65 | 74.03 ± 10.23 | <0.001 |
| BMI, kg/m2 | 23.37 ± 3.53 | 20.80 ± 2.01 | 25.71 ± 2.55 | 20.71 ± 2.02 | 26.55 ± 3.03 | <0.001 |
| Rural residence, n (%) | 4417 (67.6) | 1904 (73.8) | 1435 (62.9) | 504 (73.5) | 574 (58.2) | <0.001 |
| Region* , n (%) |  |  |  |  |  | <0.001 |
| North | 2831 (43.3) | 975 (37.8) | 1168 (51.2) | 222 (32.4) | 466 (47.3) |  |
| South | 3703 (56.7) | 1606 (62.2) | 1113 (48.8) | 464 (67.6) | 520 (52.7) |  |
| Education, n (%) |  |  |  |  |  | 0.080 |
| Junior high school and below | 5928 (90.7) | 2374 (92.0) | 2041 (89.5) | 626 (91.3) | 887 (90.0) |  |
| Senior high school | 552 (8.4) | 185 (7.2) | 221 (9.7) | 56 (8.2) | 90 (9.1) |  |
| Tertiary | 54 (0.8) | 22 (0.9) | 19 (0.8) | 4 (0.6) | 9 (0.9) |  |
| Marital status, n (%) |  |  |  |  |  |  |
| Married and living with spouse | 5571 (85.3) | 2167 (84.0) | 2011 (88.2) | 546 (79.6) | 847 (85.9) | <0.001 |
| Others | 963 (14.7) | 414 (16.0) | 270 (11.8) | 140 (20.4) | 139 (14.1) | <0.001 |
| Alcohol consumption, n (%) | 2729 (41.8) | 1134 (43.9) | 915 (40.1) | 314 (45.8) | 366 (37.1) | <0.001 |
| Smoking, n (%) | 2521 (38.6) | 1094 (42.4) | 731 (32.0) | 370 (53.9) | 326 (33.1) | <0.001 |
| Hemoglobin^&^, g/dL | 14.36 ± 2.20 | 14.16 ± 2.13 | 14.52 ± 2.17 | 14.34 ± 2.49 | 14.57 ± 2.14 | <0.001 |
| FBG, mg/dL | 109.37 ± 35.00 | 101.22 ± 21.05 | 114.75 ± 37.34 | 103.34 ± 27.13 | 122.41 ± 52.82 | <0.001 |
| HbA1c^&^, % | 5.25 ± 0.79 | 5.12 ± 0.57 | 5.32 ±0.87 | 5.15 ± 0.57 | 5.53 ± 1.08 | <0.001 |
| hsCRP, mg/L | 0.98 (0.53−2.06) | 0.61 (0.39−0.98) | 0.84 (0.55−1.27) | 3.95 (2.69−7.46) | 3.48 (2.54−5.54) | <0.001 |
| TC, mg/dL | 193.96 ± 38.62 | 189.05 ± 35.74 | 199.26 ± 39.36 | 182.21 ± 36.51 | 202.73 ± 41.63 | <0.001 |
| TG, mg/dl | 132.71 ± 112.56 | 90.78 ± 42.70 | 171.71 ± 143.52 | 91.97 ± 45.42 | 180.61 ± 136.23 | <0.001 |
| HDL, mg/dL | 51.56 ± 15.30 | 58.18 ± 15.12 | 46.40 ± 12.96 | 54.86 ± 15.27 | 43.89 ± 12.64 | <0.001 |
| LDL^&^, mg/dL | 116.62 ± 34.74 | 114.81 ± 31.81 | 118.68 ± 35.98 | 110.12 ± 32.70 | 121.13 ± 39.33 | <0.001 |
| BUN^&^, mg/dL | 15.71 ± 4.42 | 16.04 ± 4.50 | 15.45 ± 4.21 | 16.00 ± 5.04 | 15.21 ± 4.15 | <0.001 |
| UA, mg/dL | 4.41 ± 1.22 | 4.21 ± 1.15 | 4.44 ± 1.22 | 4.47 ± 1.18 | 4.81 ± 1.29 | <0.001 |
| Serum creatinine^&^, mg/dL | 0.77 ± 0.19 | 0.77 ± 0.18 | 0.76 ± 0.18 | 0.79 ± 0.21 | 0.78 ± 0.20 | 0.002 |
| Hypertension, n (%) | 2474 (37.9) | 679 (26.3) | 1031 (45.2) | 241 (35.1) | 523 (53.0) | <0.001 |
| Kidney disease^&^, n (%) | 313 (4.8) | 132 (5.2) | 104 (4.6) | 33 (4.8) | 44 (4.5) | 0.768 |
| Dyslipidemia, n (%) | 459 (7.2) | 86 (3.4) | 224 (10.0) | 15 (2.2) | 134 (14.0) | <0.001 |
| DM^&^, n (%) | 315 (4.9) | 56 (2.2) | 152 (6.7) | 13 (1.9) | 94 (9.7) | <0.001 |

BMI, body mass index; BUN, blood urea nitrogen; DBP, diastolic blood pressure; DM, diabetes mellitus; FBG, fasting blood glucose; HbA1c, glycosylated hemoglobin A1c; HDL, high density lipoprotein; hsCRP, hypersensitive C-reactive protein; LDL, low density lipoprotein; SBP, systolic blood pressure; TC, total cholesterol; TG, triglycerides; TyG-BMI, triglyceride glucose-body mass index; UA, uric acid

*Region was divided into north and south based on the Qinling Mountains-Huaihe River Line

&data for some participants were missing

**Table S6.** Baseline characteristics of participants with or without CVD

| **Characteristics** | **Overall**  **(n=6534)** | **Without CVD**  **(n=5046)** | **CVD**  **(n=1488)** | ***P* value** |
| --- | --- | --- | --- | --- |
| Age, years | 58.33 ± 8.80 | 57.71 ± 8.69 | 60.42 ± 8.83 | <0.001 |
| Female, n (%) | 3529 (54.0) | 2662 (52.8) | 867 (58.3) | <0.001 |
| SBP^&^, mmHg | 128.37 ± 20.75 | 126.97 ± 20.17 | 133.14 ± 21.96 | <0.001 |
| DBP^&^, mmHg | 74.95 ± 12.03 | 74.43 ± 11.80 | 76.74 ± 12.61 | <0.001 |
| Heart rate^&^, rpm | 72.05 ± 10.20 | 71.83 ± 10.07 | 72.80 ± 10.58 | 0.001 |
| BMI, kg/m2 | 23.37 ± 3.53 | 23.20 ± 3.43 | 23.97 ± 3.78 | <0.001 |
| Rural residence, n (%) | 4417 (67.6) | 3431 (68.0) | 986 (66.3) | 0.210 |
| Region* , n (%) |  |  |  | <0.001 |
| North | 2831 (43.3) | 1984 (39.3) | 847 (56.9) |  |
| South | 3703 (56.7) | 3062 (60.7) | 641 (43.1) |  |
| Education, n (%) |  |  |  | 0.273 |
| Junior high school and below | 5928 (90.7) | 4587 (90.9) | 1341 (90.1) |  |
| Senior high school | 552 (8.4) | 422 (8.4) | 130 (8.7) |  |
| Tertiary | 54 (0.8) | 37 (0.7) | 17 (1.1) |  |
| Marital status, n (%) |  |  |  | 0.002 |
| Married and living with spouse | 5571 (85.3) | 4339 (86.0) | 1232 (82.8) |  |
| Others | 963 (14.7) | 707 (14.0) | 256 (17.2) |  |
| Alcohol consumption, n (%) | 2729 (41.8) | 2141 (42.4) | 588 (39.5) | 0.045 |
| Smoking, n (%) | 2521 (38.6) | 1964 (38.9) | 557 (37.4) | 0.300 |
| Hemoglobin^&^, g/dL | 14.36 ± 2.20 | 14.35 ± 2.20 | 14.41 ± 2.18 | 0.384 |
| FBG, mg/dL | 109.37 ± 35.00 | 108.53 ± 33.01 | 112.22 ± 40.92 | <0.001 |
| HbA1c^&^, % | 5.25 ± 0.79 | 5.23 ± 0.75 | 5.33 ± 0.91 | <0.001 |
| hsCRP, mg/L | 0.98 (0.53−2.06) | 0.94 (0.52−1.94) | 1.14 (0.59−2.32) | <0.001 |
| TC, mg/dL | 193.96 ± 38.62 | 193.37 ± 38.78 | 195.97 ± 38.03 | 0.022 |
| TG, mg/dl | 132.71 ± 112.56 | 131.32 ± 114.95 | 137.44 ± 103.94 | 0.065 |
| HDL, mg/dL | 51.56 ± 15.30 | 51.83 ± 15.30 | 50.64 ± 15.28 | 0.008 |
| LDL^&^, mg/dL | 116.62 ± 34.74 | 116.09 ± 34.51 | 118.41 ± 35.47 | 0.023 |
| BUN^&^, mg/dL | 15.71 ± 4.42 | 15.77 ± 4.42 | 15.50 ± 4.43 | 0.039 |
| UA, mg/dL | 4.41 ± 1.22 | 4.41 ± 1.21 | 4.39 ± 1.25 | 0.443 |
| Serum creatinine^&^, mg/dL | 0.77 ± 0.19 | 0.77 ± 0.18 | 0.77 ± 0.19 | 0.806 |
| Hypertension, n (%) | 2474 (37.9) | 1722 (34.1) | 752 (50.5) | <0.001 |
| Kidney disease^&^, n (%) | 313 (4.8) | 215 (4.3) | 98 (6.6) | <0.001 |
| Dyslipidemia, n (%) | 459 (7.2) | 264 (5.3) | 195 (13.4) | <0.001 |
| DM^&^, n (%) | 315 (4.9) | 206 (4.1) | 109 (7.4) | <0.001 |

BMI, body mass index; BUN, blood urea nitrogen; DBP, diastolic blood pressure; DM, diabetes mellitus; FBG, fasting blood glucose; HbA1c, glycosylated hemoglobin A1c; HDL, high density lipoprotein; hsCRP, hypersensitive C-reactive protein; LDL, low density lipoprotein; SBP, systolic blood pressure; TC, total cholesterol; TG, triglycerides; TyG-BMI, triglyceride glucose-body mass index; UA, uric acid

*Region was divided into north and south based on the Qinling Mountains-Huaihe River Line

&data for some participants were missing

**Table S7.** Baseline characteristics of patients undergoing percutaneous coronary intervention by TyG-BMI.

| **Characteristics** | **Overall**  **(n=805)** | **Low TyG-BMI (n=402)** | **High TyG-BMI (n=403)** | ***P* value** |
| --- | --- | --- | --- | --- |
| Demographics |  |  |  |  |
| Age, years | 68.25 ± 6.15 | 68.43 ± 6.23 | 68.08 ± 6.08 | 0.429 |
| Male, (%) | 485 (60.2) | 306 (76.1) | 179 (44.4) | <0.001 |
| Smoking, n (%) | 220 (27.3) | 131 (32.6) | 89 (22.1) | 0.001 |
| BMI, kg/m^2^ | 24.19 ± 3.84 | 21.47 ± 2.27 | 26.90 ± 3.09 | <0.001 |
| Laboratory data |  |  |  |  |
| Glycemia, mmol/L | 6.11 ± 2.45 | 5.52 ± 1.62 | 6.70 ± 2.95 | <0.001 |
| Creatinine, μmol/L | 73.70 ± 31.87 | 75.29 ± 25.45 | 72.12 ± 37.15 | 0.158 |
| Uric acid, μmol/L | 305.73 ± 104.20 | 309.00 ± 97.10 | 302.47 ± 110.87 | 0.375 |
| TG, mmol/L | 1.83 ± 1.37 | 1.46 ± 0.75 | 2.21 ± 1.70 | <0.001 |
| TC, mmol/L | 4.29 ± 1.03 | 4.10 ± 0.92 | 4.47 ± 1.09 | <0.001 |
| HDL-C, mmol/L | 1.12 ± 0.35 | 1.15 ± 0.36 | 1.09 ± 0.34 | 0.02 |
| LDL-C, mmol/L | 2.73 ± 0.92 | 2.64 ± 0.89 | 2.82 ± 0.94 | 0.005 |
| Medical history |  |  |  |  |
| Heart failure, n (%) | 101 (12.5) | 50 (12.4) | 51 (12.7) | 0.999 |
| Atrial fibrillation, n (%) | 21 (2.6) | 14 (3.5) | 7 (1.7) | 0.183 |
| Previous AMI, n (%) | 79 (9.8) | 46 (11.4) | 33 (8.2) | 0.152 |
| Previous PCI, n (%) | 51 (6.3) | 27 (6.7) | 24 (6.0) | 0.765 |
| Hypertension, n (%) | 422 (52.4) | 169 (42.0) | 253 (62.8) | <0.001 |
| Diabetes mellitus, n (%) | 212 (26.3) | 80 (19.9) | 132 (32.8) | <0.001 |
| Treatment |  |  |  |  |
| Aspirin, n (%) | 793 (98.5) | 400 (99.5) | 393 (97.5) | 0.042 |
| Clopidogrel, n (%) | 772 (95.9) | 380 (94.5) | 392 (97.3) | 0.091 |
| Beta blocker, n (%) | 537 (66.7) | 265 (65.9) | 272 (67.5) | 0.69 |
| ACEI, n (%) | 456 (56.6) | 208 (51.7) | 248 (61.5) | 0.006 |
| CCB, n (%) | 202 (25.1) | 74 (18.4) | 128 (31.8) | <0.001 |
| Statin, n (%) | 755 (93.8) | 379 (94.3) | 376 (93.3) | 0.668 |
| Number of diseased vessels |  |  |  | 0.594 |
| 1-vessel disease, n (%) | 239 (29.7) | 119 (29.6) | 120 (29.8) |  |
| 2-vessel disease, n (%) | 324 (40.2) | 168 (41.8) | 156 (38.7) |  |
| ≥3-vessel disease, n (%) | 242 (30.1) | 115 (28.6) | 127 (31.5) |  |
| Location of target lesions |  |  |  |  |
| LM, n (%) | 33 (4.1) | 20 (5.0) | 13 (3.2) | 0.283 |
| LAD, n (%) | 689 (85.6) | 344 (85.6) | 345 (85.6) | 0.996 |
| LCX, n (%) | 430 (53.4) | 213 (53.0) | 217 (53.8) | 0.862 |
| RCA, n (%) | 450 (55.9) | 221 (55.0) | 229 (56.8) | 0.648 |
| Characteristics of lesions |  |  |  |  |
| Occlusion, n (%) | 101 (12.5) | 46 (11.4) | 55 (13.6) | 0.402 |
| CTO, n (%) | 77 (9.6) | 39 (9.7) | 38 (9.4) | 0.991 |
| Ostial lesion, n (%) | 97 (12.0) | 55 (13.7) | 42 (10.4) | 0.189 |
| Bifurcation lesion, n (%) | 140 (17.4) | 74 (18.4) | 66 (16.4) | 0.505 |
| Number of treated vessels |  |  |  | 0.022 |
| 1-vessel disease, n (%) | 431 (53.5) | 233 (58.0) | 198 (49.1) |  |
| 2-vessel disease, n (%) | 289 (35.9) | 126 (31.3) | 163 (40.4) |  |
| ≥3-vessel disease, n (%) | 85 (10.6) | 43 (10.7) | 42 (10.4) |  |
| Length of stents, (mm) | 51.51 ± 31.36 | 49.24 ± 29.87 | 53.78 ± 32.67 | 0.04 |
| Diameter of stents, (mm) | 3.06 ± 1.03 | 3.03 ± 0.42 | 3.09 ± 1.40 | 0.418 |

**Table S8**. Joint association of TyG-BMI and hsCRP with new-onset CVD, stroke and cardiac events after excluding individuals experienced CVD during or before Survey 2

| **Variables** | **Model 1** | | **Model 2** | | **Model 3** | |
| --- | --- | --- | --- | --- | --- | --- |
|  | HR (95% CI) | *P* value | HR (95% CI) | *P* value | HR (95% CI) | *P* value |
| CVD |  |  |  |  |  |  |
| Group 1 | Ref |  | Ref |  | Ref |  |
| Group 2 | 1.25 (1.09−1.42) | 0.001 | 1.21 (1.05−1.38) | 0.006 | 1.12 (0.97−1.30) | 0.109 |
| Group 3 | 1.12 (0.91−1.37) | 0.280 | 1.05 (0.86−1.29) | 0.627 | 1.05 (0.86−1.30) | 0.625 |
| Group 4 | 1.56 (1.34−1.83) | <0.001 | 1.43 (1.22−1.69) | <0.001 | 1.29 (1.08−1.54) | 0.005 |
| Stroke |  |  |  |  |  |  |
| Group 1 | Ref |  | Ref |  | Ref |  |
| Group 2 | 1.56 (1.23−1.97) | <0.001 | 1.43 (1.12−1.82) | 0.004 | 1.34 (1.04−1.73) | 0.026 |
| Group 3 | 1.70 (1.22−2.36) | 0.002 | 1.50 (1.08−2.08) | 0.016 | 1.50 (1.07−2.10) | 0.018 |
| Group 4 | 2.00 (1.52−2.63) | <0.001 | 1.72 (1.29−2.28) | <0.001 | 1.54 (1.14−2.09) | 0.005 |
| Cardiac events |  |  |  |  |  |  |
| Group 1 | Ref |  | Ref |  | Ref |  |
| Group 2 | 1.19 (1.02−1.38) | 0.025 | 1.17 (1.00−1.37) | 0.046 | 1.09 (0.92−1.29) | 0.301 |
| Group 3 | 0.92 (0.72−1.18) | 0.511 | 0.89 (0.70−1.14) | 0.366 | 0.89 (0.69−1.15) | 0.381 |
| Group 4 | 1.49 (1.24−1.79) | <0.001 | 1.40 (1.16−1.69) | <0.001 | 1.26 (1.02−1.54) | 0.029 |

Group 1 is low TyG-BMI and low hsCRP; Group 2 is high TyG-BMI and low hsCRP; Group 3 is low TyG-BMI and high hsCRP; Group 4 is high TyG-BMI and high hsCRP.

Model 1: unadjusted

Model 2: adjusted for age, sex, SBP and DBP

Model 3: model 2 + further adjusted for marital status, education, living place, serum creatine, hemoglobin, uric acid, BUN, TC, LDL-C, hypertension, dyslipidemia, DM, smoking status and alcohol consumption status

**Table S9.** Joint association of TyG-BMI and hsCRP with new-onset CVD, stroke and cardiac events after excluding individuals with extremely high TyG-BMI or hsCRP (>99% percentile).

| **Variables** | **Model 1** | | **Model 2** | | **Model 3** | |
| --- | --- | --- | --- | --- | --- | --- |
|  | HR (95% CI) | *P* value | HR (95% CI) | *P* value | HR (95% CI) | *P* value |
| CVD |  |  |  |  |  |  |
| Group 1 | Ref |  | Ref |  | Ref |  |
| Group 2 | 1.25 (1.10−1.41) | <0.001 | 1.20 (1.06−1.36) | 0.005 | 1.13 (0.99−1.29) | 0.081 |
| Group 3 | 1.21 (1.00−1.45) | 0.050 | 1.14 (0.94−1.37) | 0.183 | 1.13 (0.93−1.37) | 0.223 |
| Group 4 | 1.62 (1.40−1.88) | <0.001 | 1.48 (1.27−1.73) | <0.001 | 1.37 (1.16−1.61) | <0.001 |
| Stroke |  |  |  |  |  |  |
| Group 1 | Ref |  | Ref |  | Ref |  |
| Group 2 | 1.61 (1.29−2.02) | <0.001 | 1.48 (1.17−1.86) | 0.001 | 1.39 (1.09−1.78) | 0.008 |
| Group 3 | 1.85 (1.35−2.53) | <0.001 | 1.61 (1.18−2.21) | 0.003 | 1.59 (1.16−2.19) | 0.004 |
| Group 4 | 2.04 (1.56−2.66) | <0.001 | 1.76 (1.34−2.32) | <0.001 | 1.58 (1.18−2.12) | 0.002 |
| Cardiac events |  |  |  |  |  |  |
| Group 1 | Ref |  | Ref |  | Ref |  |
| Group 2 | 1.18 (1.02−1.36) | 0.018 | 1.16 (1.00−1.34) | 0.045 | 1.08 (0.93−1.26) | 0.335 |
| Group 3 | 1.06 (0.85−1.32) | 0.604 | 1.03 (0.82−1.28) | 0.816 | 1.02 (0.81−1.28) | 0.862 |
| Group 4 | 1.55 (1.31−1.84) | <0.001 | 1.45 (1.22−1.73) | <0.001 | 1.33 (1.11−1.61) | 0.003 |

Group 1 is low TyG-BMI and low hsCRP; Group 2 is high TyG-BMI and low hsCRP; Group 3 is low TyG-BMI and high hsCRP; Group 4 is high TyG-BMI and high hsCRP.

Model 1: unadjusted

Model 2: adjusted for age, sex, SBP and DBP

Model 3: model 2 + further adjusted for marital status, education, living place, serum creatine, hemoglobin, uric acid, BUN, TC, LDL-C, hypertension, dyslipidemia, DM, smoking status and alcohol consumption status

**Table S10.** Joint association of TyG-BMI and hsCRP with new-onset CVD, stroke and cardiac events after excluding individuals with diabetes mellitus

| **Variables** | **Model 1** | | **Model 2** | | **Model 3** | |
| --- | --- | --- | --- | --- | --- | --- |
|  | HR (95% CI) | *P* value | HR (95% CI) | *P* value | HR (95% CI) | *P* value |
| CVD |  |  |  |  |  |  |
| Group 1 | Ref |  | Ref |  | Ref |  |
| Group 2 | 1.20 (1.06−1.37) | 0.004 | 1.16 (1.02−1.33) | 0.023 | 1.10 (0.96−1.27) | 0.157 |
| Group 3 | 1.20 (1.00−1.45) | 0.052 | 1.13 (0.94−1.37) | 0.193 | 1.11 (0.91−1.34) | 0.309 |
| Group 4 | 1.60 (1.38−1.86) | <0.001 | 1.47 (1.26−1.72) | <0.001 | 1.37 (1.16−1.62) | <0.001 |
| Stroke |  |  |  |  |  |  |
| Group 1 | Ref |  | Ref |  | Ref |  |
| Group 2 | 1.52 (1.21−1.91) | <0.001 | 1.39 (1.10−1.77) | 0.006 | 1.34 (1.04−1.71) | 0.023 |
| Group 3 | 1.76 (1.29−2.41) | <0.001 | 1.54 (1.12−2.11) | 0.007 | 1.51 (1.10−2.08) | 0.011 |
| Group 4 | 1.97 (1.50−2.58) | <0.001 | 1.71 (1.30−2.26) | <0.001 | 1.58 (1.17−2.12) | 0.002 |
| Cardiac events |  |  |  |  |  |  |
| Group 1 | Ref |  | Ref |  | Ref |  |
| Group 2 | 1.14 (0.99−1.32) | 0.072 | 1.13 (0.97−1.31) | 0.119 | 1.07 (0.91−1.25) | 0.410 |
| Group 3 | 1.06 (0.85−1.32) | 0.601 | 1.03 (0.82−1.28) | 0.812 | 1.00 (0.79−1.26) | 0.992 |
| Group 4 | 1.53 (1.28−1.81) | <0.001 | 1.43 (1.20−1.71) | <0.001 | 1.34 (1.11−1.63) | 0.002 |

Group 1 is low TyG-BMI and low hsCRP; Group 2 is high TyG-BMI and low hsCRP; Group 3 is low TyG-BMI and high hsCRP; Group 4 is high TyG-BMI and high hsCRP.

Model 1: unadjusted

Model 2: adjusted for age, sex, SBP and DBP

Model 3: model 2 + further adjusted for marital status, education, living place, serum creatine, hemoglobin, uric acid, BUN, TC, LDL-C, hypertension, dyslipidemia, DM, smoking status and alcohol consumption status

**Table S11.** Joint association of TyG-BMI and hsCRP with new-onset CVD, stroke and cardiac events after imputing the baseline missing values.

| **Variables** | **Model 1** | | **Model 2** | | **Model 3** | |
| --- | --- | --- | --- | --- | --- | --- |
|  | HR (95% CI) | *P* value | HR (95% CI) | *P* value | HR (95% CI) | *P* value |
| CVD |  |  |  |  |  |  |
| Group 1 | Ref |  | Ref |  | Ref |  |
| Group 2 | 1.23 (1.09−1.39) | 0.001 | 1.18 (1.04−1.34) | 0.008 | 1.10 (0.96−1.25) | 0.155 |
| Group 3 | 1.21 (1.01−1.45) | 0.043 | 1.12 (0.93−1.34) | 0.228 | 1.10 (0.92−1.33) | 0.290 |
| Group 4 | 1.65 (1.42−1.90) | <0.001 | 1.49 (1.28−1.72) | <0.001 | 1.32 (1.13−1.55) | 0.001 |
| Stroke |  |  |  |  |  |  |
| Group 1 | Ref |  | Ref |  | Ref |  |
| Group 2 | 1.60 (1.28−2.00) | <0.001 | 1.50 (1.19−1.89) | <0.001 | 1.40 (1.11−1.78) | 0.005 |
| Group 3 | 1.85 (1.37−2.50) | <0.001 | 1.61 (1.19−2.18) | 0.002 | 1.58 (1.17−2.15) | 0.003 |
| Group 4 | 2.10 (1.62−2.72) | <0.001 | 1.81 (1.39−2.36) | <0.001 | 1.60 (1.21−2.11) | 0.001 |
| Cardiac events |  |  |  |  |  |  |
| Group 1 | Ref |  | Ref |  | Ref |  |
| Group 2 | 1.16 (1.01−1.33) | 0.037 | 1.13 (0.98−1.30) | 0.090 | 1.04 (0.90−1.21) | 0.586 |
| Group 3 | 1.04 (0.84−1.29) | 0.728 | 0.99 (0.80−1.23) | 0.929 | 0.98 (0.79−1.22) | 0.853 |
| Group 4 | 1.56 (1.33−1.84) | <0.001 | 1.44 (1.22−1.70) | <0.001 | 1.27 (1.06−1.52) | 0.008 |

Group 1 is low TyG-BMI and low hsCRP; Group 2 is high TyG-BMI and low hsCRP; Group 3 is low TyG-BMI and high hsCRP; Group 4 is high TyG-BMI and high hsCRP.

Model 1: unadjusted

Model 2: adjusted for age, sex, SBP and DBP

Model 3: model 2 + further adjusted for marital status, education, living place, serum creatine, hemoglobin, uric acid, BUN, TC, LDL-C, hypertension, dyslipidemia, DM, smoking status and alcohol consumption status


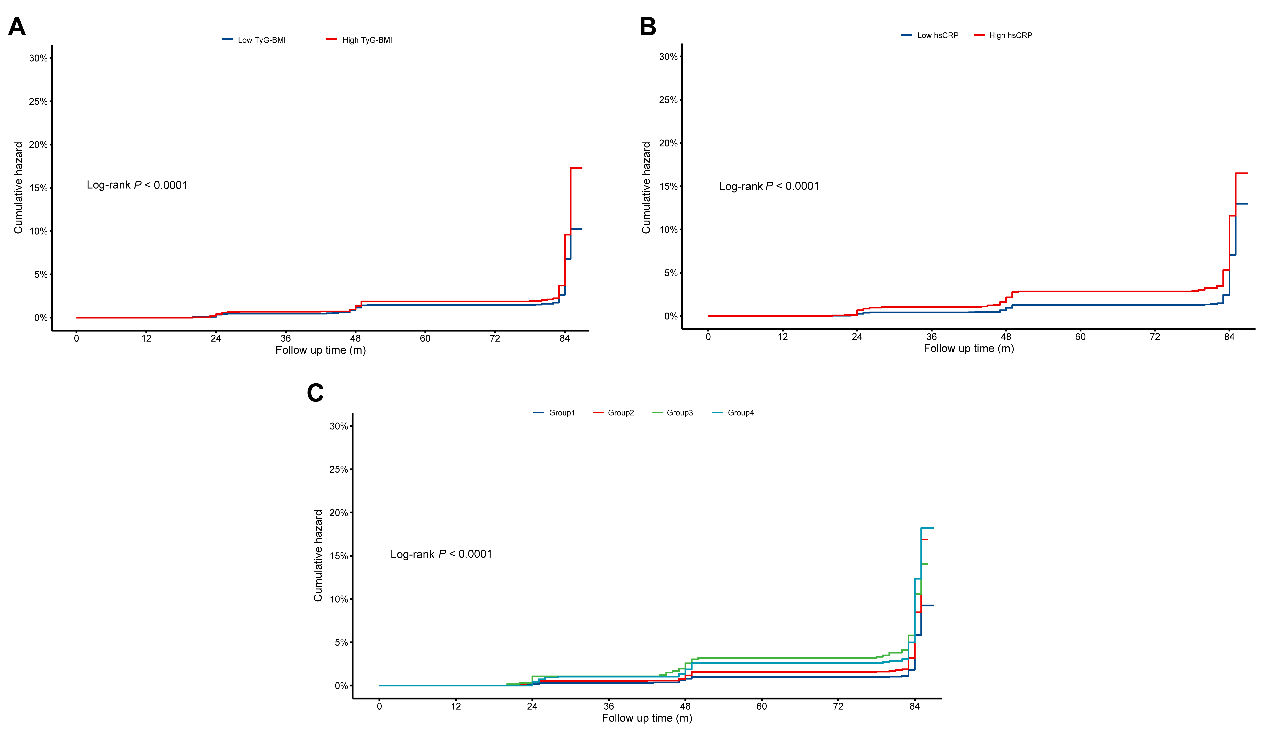


**Figure S1.** Kaplan–Meier curves for the cumulative incidence of stroke according to the TyG-BMI index (A), hsCRP (B), or across TyG-BMI index and hsCRP groups (C). Group 1 is low TyG-BMI and low hsCRP; Group 2 is high TyG-BMI and low hsCRP; Group 3 is low TyG-BMI and high hsCRP; Group 4 is high TyG-BMI and high hsCRP.


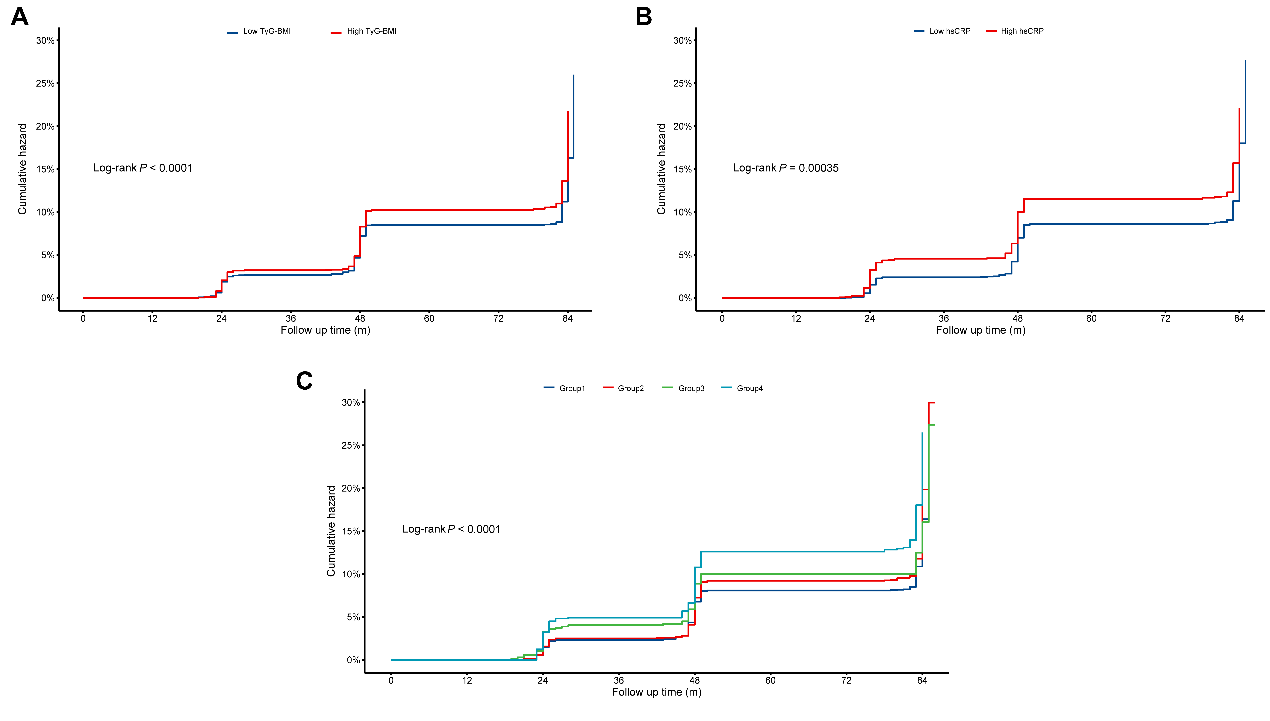


**Figure S2.** Kaplan–Meier curves for the cumulative incidence of cardiac events according to the TyG-BMI index (A), hsCRP (B), or across TyG-BMI index and hsCRP groups (C). Group 1 is low TyG-BMI and low hsCRP; Group 2 is high TyG-BMI and low hsCRP; Group 3 is low TyG-BMI and high hsCRP; Group 4 is high TyG-BMI and high hsCRP.


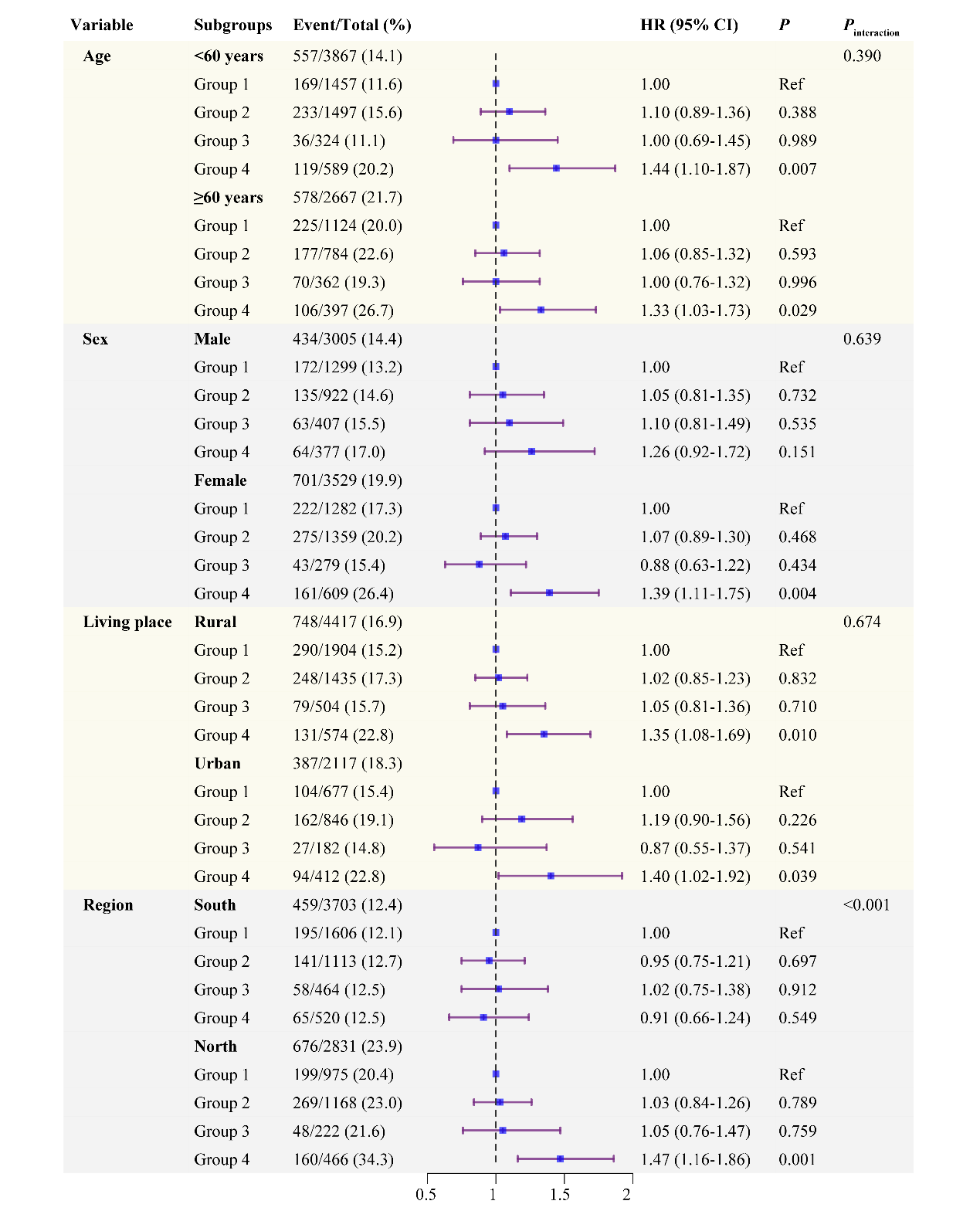


**Figure S3.** Subgroup and interaction analyses between the four groups and cardiac events across various subgroups. Group 1 is low TyG-BMI and low hsCRP; Group 2 is high TyG-BMI and low hsCRP; Group 3 is low TyG-BMI and high hsCRP; Group 4 is high TyG-BMI and high hsCRP.


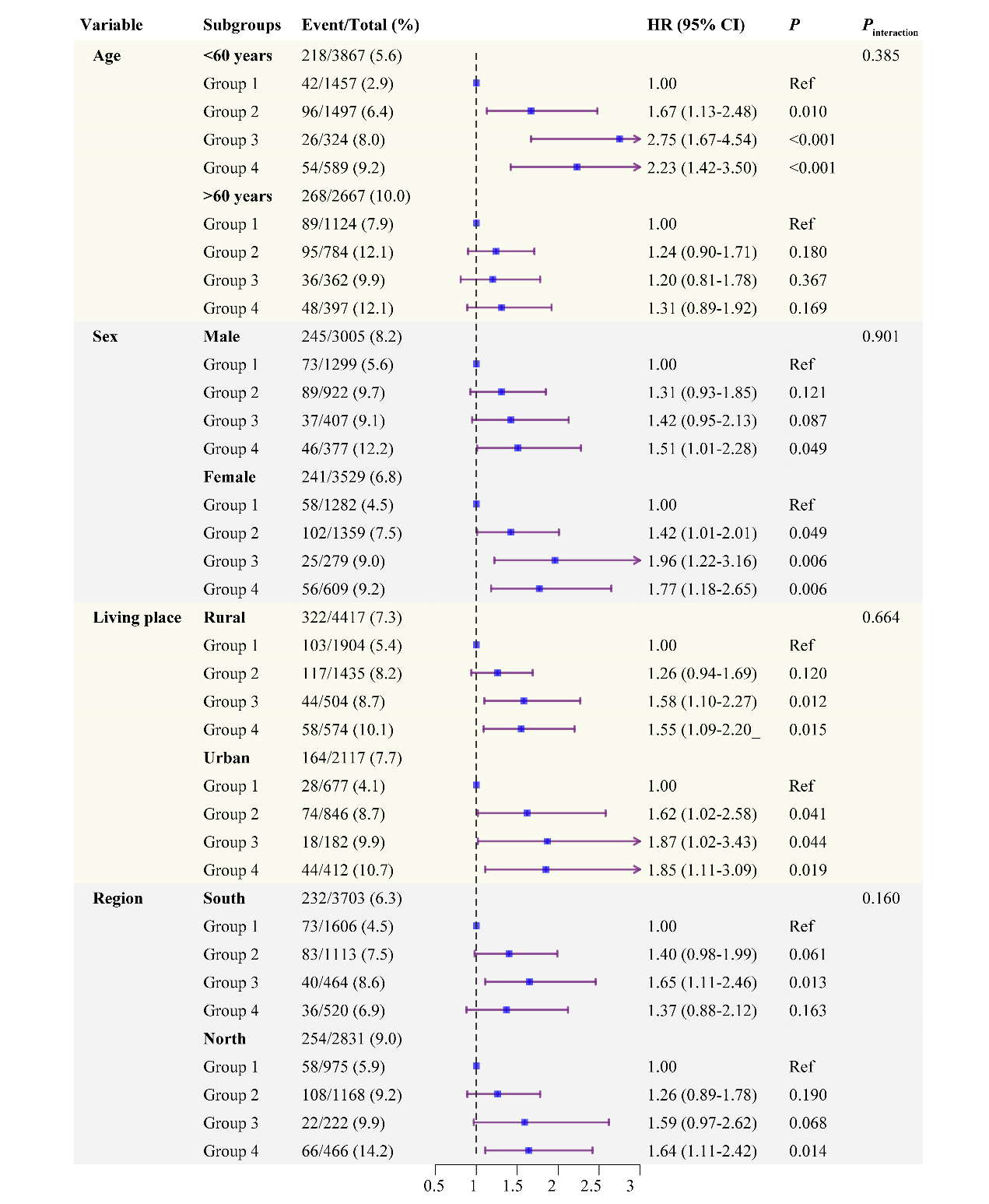


**Figure S4.** Subgroup and interaction analyses between the four groups and stroke across various subgroups. Group 1 is low TyG-BMI and low hsCRP; Group 2 is high TyG-BMI and low hsCRP; Group 3 is low TyG-BMI and high hsCRP; Group 4 is high TyG-BMI and high hsCRP.


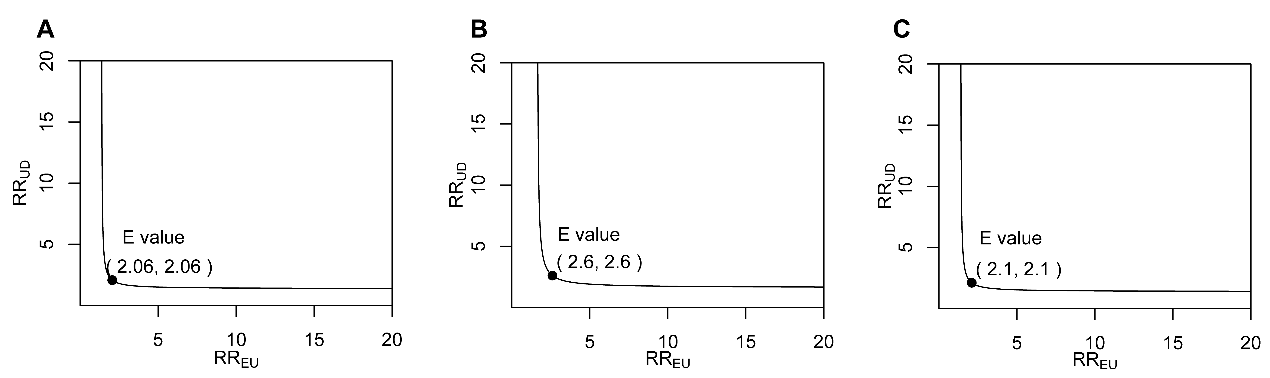


**Figure S5.** E-value analysis to evaluate the extent of unmeasured confounders that would be required to negate the observed results. (A) cardiac events; (B) stroke; (C) cardiovascular disease.

**Reference**

1. Zhao Y, Hu Y, Smith JP, Strauss J, Yang G: **Cohort profile: the China Health and Retirement Longitudinal Study (CHARLS)**. *Int J Epidemiol* 2014, **43**(1):61-68.

2. Cui C, Liu L, Zhang T, Fang L, Mo Z, Qi Y, Zheng J, Wang Z, Xu H, Yan H *et al*: **Triglyceride-glucose index, renal function and cardiovascular disease: a national cohort study**. *Cardiovasc Diabetol* 2023, **22**(1):325.

3. Lin L, Wang HH, Liu Y, Lu C, Chen W, Guo VY: **Indoor solid fuel use for heating and cooking with blood pressure and hypertension: A cross-sectional study among middle-aged and older adults in China**. *Indoor Air* 2021, **31**(6):2158-2166.

4. Williams B, Mancia G, Spiering W, Agabiti Rosei E, Azizi M, Burnier M, Clement DL, Coca A, de Simone G, Dominiczak A *et al*: **2018 ESC/ESH Guidelines for the management of arterial hypertension**. *Eur Heart J* 2018, **39**(33):3021-3104.

5. Yu J, Yi Q, Chen G, Hou L, Liu Q, Xu Y, Qiu Y, Song P: **The visceral adiposity index and risk of type 2 diabetes mellitus in China: A national cohort analysis**. *Diabetes Metab Res Rev* 2022, **38**(3):e3507.

6. Zheng X, Han L, Shen S: **Hypertension, remnant cholesterol and cardiovascular disease: evidence from the China health and retirement longitudinal study**. *Journal of Hypertension* 2022, **40**(11):2292-2298.

7. VanderWeele TJ, Ding P: **Sensitivity Analysis in Observational Research: Introducing the E-Value**. *Ann Intern Med* 2017, **167**(4):268-274.
